# Immature Dentate Granule Cells Require *Ntrk2/Trkb* for the Formation of Functional Hippocampal Circuitry

**DOI:** 10.1016/j.isci.2020.101078

**Published:** 2020-04-18

**Authors:** Sylvia Badurek, Marilena Griguoli, Aman Asif-Malik, Barbara Zonta, Fei Guo, Silvia Middei, Laura Lagostena, Maria Teresa Jurado-Parras, Thomas H. Gillingwater, Agnès Gruart, José María Delgado-García, Enrico Cherubini, Liliana Minichiello

**Affiliations:** 1 Department of Pharmacology, University of Oxford, Oxford, United Kingdom; 2 Centre for Neuroregeneration, University of Edinburgh, Edinburgh, United Kingdom; 3 European Molecular Biology Laboratory, Mouse Biology Unit, Monterotondo, Rome, Italy; 4 European Brain Research Institute, Rome, Italy; 5 Institute of Cell Biology and Neurobiology, National Research Council, Monterotondo, Rome, Italy; 6 International School for Advanced Studies (SISSA), Department of Neuroscience, Trieste, Italy; 7 Division of Neurosciences, University Pablo de Olavide, Seville, Spain; 8 Biomedical Sciences, Edinburgh Medical School, University of Edinburgh, Edinburgh, United Kingdom

**Keywords:** Genetics, Neuroscience

## Abstract

Early in brain development, impaired neuronal signaling during time-sensitive windows triggers the onset of neurodevelopmental disorders. GABA, through its depolarizing and excitatory actions, drives early developmental events including neuronal circuit formation and refinement. BDNF/TrkB signaling cooperates with GABA actions. How these developmental processes influence the formation of neural circuits and affect adult brain function is unknown. Here, we show that early deletion of *Ntrk2/Trkb* from immature mouse hippocampal dentate granule cells (DGCs) affects the integration and maturation of newly formed DGCs in the hippocampal circuitry and drives a premature shift from depolarizing to hyperpolarizing GABAergic actions in the target of DGCs, the CA3 principal cells of the hippocampus, by reducing the expression of the cation-chloride importer *Nkcc1*. These changes lead to the disruption of early synchronized neuronal activity at the network level and impaired morphological maturation of CA3 pyramidal neurons, ultimately contributing to altered adult hippocampal synaptic plasticity and cognitive processes.

## Highlights

• TrkB signaling shapes hippocampal circuitry by controlling early GABA signaling
• Altered *Nkcc1* expression reduces CA3 pyramidal cell GABAergic excitatory drive
• Early network activity is crucial for establishing functional hippocampal circuitry

## Graphical Abstract

**Figure:**
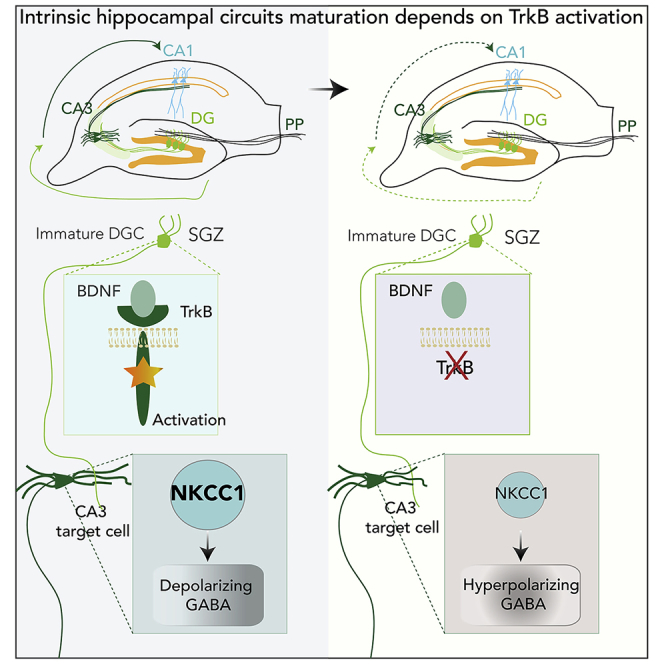

## Introduction

Early in postnatal development, experience shapes neuronal circuits during time-sensitive periods. This process affects adult brain functions and leads to neurodevelopmental disorders arising from early dysfunction of neuronal signaling (Meredith et al., 2012). The hippocampus is a brain structure involved in several higher brain functions, including learning and memory, and spatial coding. The dentate gyrus (DG), input region of the hippocampus, plays a crucial role in these functions. Most dentate granule cells (DGCs) in the rodent brain are generated in the first postnatal week. The adult structure of the rodent DG, including the subgranular zone (SGZ), where neurons are continuously generated, is established between postnatal days (P) 7 and 14 (Nicola et al., 2015). Latest discoveries suggest that developmental and adult dentate neurogenesis are one continuous process whereby during embryonic development, dentate neural precursors generate DGCs to establish the primitive DG. They then adopt adult radial glial-like capacities in the SGZ during the early postnatal period and continue to generate DGCs in the adult thus resembling a unified process of extended development (Berg et al., 2019, Hochgerner et al., 2018).

Activity-dependent GABA-mediated excitation in the adult brain drives hippocampal progenitor cells from proliferation to neuroblast, migration, and integration of newly generated neurons in pre-existing functional circuits (Ge et al., 2006, Ge et al., 2007, Tozuka et al., 2005). This is due to the early depolarizing and excitatory effects of GABA on neural precursors and immature neurons exhibiting higher intracellular chloride concentration [Cl^–^], which results from the differential temporal expression of the cation-chloride cotransporters NKCC1 and KCC2, involved in chloride uptake and extrusion, respectively (Ben-Ari, 2002, Ben-Ari et al., 2012, Owens and Kriegstein, 2002). In postnatal development, it has been shown that the premature shift of GABA from depolarizing to hyperpolarizing direction alters the morphological maturation of neonatal cortical neurons *in vivo* (Cancedda et al., 2007). Furthermore, in the immature hippocampus, the depolarizing action of GABA contributes to generate coherent network oscillations such as giant depolarizing potentials (GDPs), which represent a primordial form of synchrony between neurons that precedes more organized forms of activity like theta and gamma rhythms. GDP-associated Ca^2+^ transients are instrumental in modifying synaptic efficacy at emerging GABAergic and glutamatergic synapses (Ben-Ari et al., 2012), contributing to the structural refinement of neuronal connectivity and the establishment of adult neural circuits. These are fundamental functions, and unsurprisingly, impaired GABAergic transmission gives rise to an array of neurodevelopmental disorders (Deidda et al., 2014). However, how these processes are triggered and influence adult brain function is unknown. GABAergic development relies highly on BDNF/TrkB signaling (Gottmann et al., 2009, Hong et al., 2008). The latter is renowned for being one of the most critical regulators of glutamatergic and GABAergic synapse development and function in the developing and adult central nervous system (Cohen-Cory et al., 2010, Lu et al., 2005, Minichiello, 2009, Musumeci et al., 2009). Early in postnatal life, BDNF/TrkB signaling is instrumental in tuning hippocampal synaptic connections, in particular, at immature mossy fiber (MF)-CA3 synapses through the activation of the MAPK/ERK cascade (Mohajerani et al., 2007, Sivakumaran et al., 2009).

In this study, we asked whether BDNF/TrkB signaling would influence the establishment of hippocampal circuitry *in vivo* and eventually animal behavior in adulthood, by affecting the early depolarizing and excitatory actions of GABA. To answer this question, we used a novel genetic mouse model to remove TrkB signaling in immature DGCs early in postnatal development, coinciding with the integration time of these cells in the hippocampal circuitry. Here we show that such deletion affects the integration and maturation of newly formed DGCs in the forming DG. This, in turn, impairs the maturation of CA3 principal neurons via reduced expression of *Nkcc1*, with a consequent premature shift of GABA action from the depolarizing to the hyperpolarizing direction and GDP disruption, ultimately resulting in altered adult hippocampal synaptic plasticity and cognitive processes.

## Results

### Conditional Removal of *Trkb* in Immature Hippocampal Granule Cells

Previously, we have shown that the BAC-*Gad1-Cre* mouse line expresses Cre-recombinase in DGCs within the hippocampal formation (Ohtsuka et al., 2013). To further characterize the hippocampal spatiotemporal expression pattern of this Cre-strain, we crossed the BAC-*Gad1-Cre* strain to different reporter lines (Z/EG, Rosa-YFP, and Rosa-Ai9-tdTomato) (Madisen et al., 2010, Novak et al., 2000). The analysis revealed *GAD1-Cre*-reporter signal at P2 in the forming DG (Figure S1A), with increasing number of recombined granule cells at P7 (Figure S1B), and in adulthood where about 70% of the total number of DAPI-stained DG nuclei were positive for the reporter signal (Figures S1E and S1H). Cell bodies in the CA3 and CA1 areas were devoid of fluorescent reporter signal in contrast with MFs, the DGCs' axons (Figure S1C, S1D, S1F, and S1G). Similarly, immunostaining for calcium-binding proteins, Parvalbumin (interneuron marker), Calretinin (interneuron and immature DGCs marker), and Calbindin (interneuron and mature DGCs marker) (von Bohlen Und Halbach, 2007) revealed no detectable overlapping staining between each primary antibody and tdTomato signal (BAC-*Gad1-Cre*^*tg/+*^; Ai9^*T/+*^) in hippocampal interneurons (Figure S1I–S1Q and insets). Colocalization was observed between tdTomato and Calretinin in immature DGCs of the SGZ (Figure S1P and inset), and consequently with Calbindin in the dentate granular cell layer (GCL) as they mature (Figure S1Q and inset). To corroborate these results, we performed immunostaining using doublecortin (DCX), a protein expressed by neuronal precursor cells and immature neurons in the adult neurogenic brain areas (Brown et al., 2003). Expression of tdTomato colocalized specifically with DCX in immature DGCs beginning to migrate and mature into the GCL (Figure S1R). These findings are in agreement with a previous study showing a similar expression pattern in a transgenic line using the GAD1 promoter to drive EGFP (Cabezas et al., 2013). Cellular localization studies of the full-length TrkB has revealed its expression in many neuronal cell types of the hippocampus, including the DGCs and the pyramidal cells of the CA3 and CA1 regions as well as interneurons (Drake et al., 1999). Importantly, a recent transcriptome analysis using single dentate neural progenitors at different stages has revealed that *Ntrk2/Trkb* expression is upregulated from embryonic (E15.5) to early postnatal (P4) and adult (P45) stage (Figure S1S, data extracted from Berg et al., 2019). Therefore, given the specificity of the BAC-*Gad1-Cre* line in immature DGCs within the hippocampus, we crossed this *Cre* strain to the *Trkb* floxed strain (Minichiello et al., 1999) generating *Trkb*^*Gad1-KO*^ mice to remove TrkB signaling from these cells (Figures S1T–S1W). This new strain allowed determining whether early GABA action requires BDNF/TrkB signaling at a critical period during development, coinciding with the integration time and maturation of newly born DGCs in the GCL, for the formation of functional hippocampal circuits. The *Trkb*^*Gad1-KO*^ mice were viable and fertile and appeared hyperactive at around 3/4 weeks of age when handling for routine husbandry procedures. It was difficult to catch by hand; mutants would be faster to escape and run around the cage. This phenotype was less evident in adulthood.

### Reduced CREB Activation and Affected Integration of Immature DGCs in Absence of TrkB Signaling

**Figure 1:**
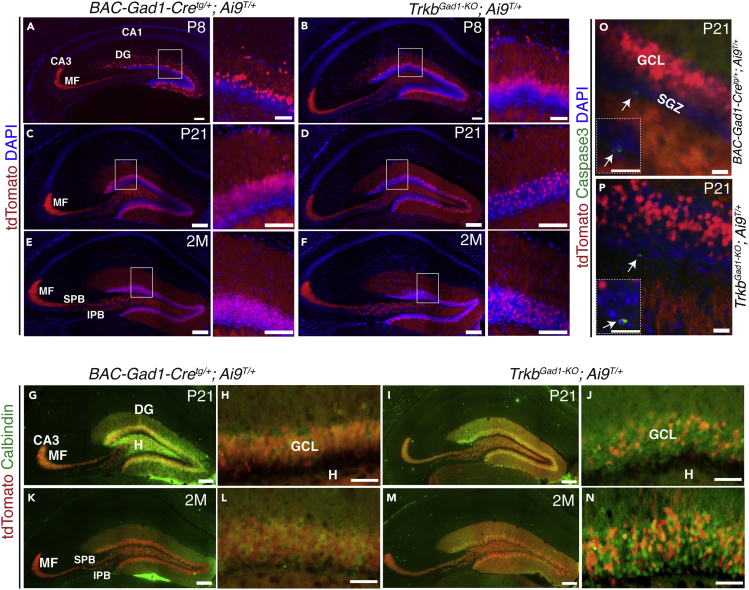
Time Course Analysis of the Dentate Gyrus Development in *Trkb*^*Gad1-KO*^ Mice and Controls (A–F) (A, C, and E) and (B, D, and F) Representative images of hippocampal coronal sections from controls (*BAC-Gad1-Cre*^*tg/+*^; *Ai9*^*T/+*^) and mutants (*Trkb*^*Gad1-KO*^; *Ai9*^*T/+*^) at postnatal days 8 and 21 and 2 months (P8, P21, and 2M), respectively, highlighting the forming dentate gyrus (DG) by tdTomato (red) endogenous fluorescence superimposed to DAPI staining (blue). Insets show higher magnification of the DG at all respective ages. Note the endogenous tdTomato fluorescence highlighting the distribution of mossy fiber bundles in control and *Trkb*^*Gad1-KO*^ mice, namely the infrapyramidal mossy fiber (IPB) and the suprapyramidal mossy fiber (SPB) bundles, as well as the mossy fiber (MF) terminals targeting the hippocampal CA3 region. (G–N) (G–J) and (K–N) Representative images of hippocampal coronal sections from controls (*BAC-Gad1-Cre*^*tg/+*^; *Ai9*^*T/+*^) and mutants *Trkb*^*Gad1-KO*^; *Ai9*^*T/+*^ at P21 and 2M, respectively, highlighting Calbindin immunostaining in mature dentate cells in the granule cell layer (GCL) and mossy fibers (MFs) targeting the CA3 region. (O and P) Representative images and respective insets showing caspase 3 immunofluorescence-stained (green) cells in the subgranular zone (SGZ) of both control (O) and mutant (P) mice indicated by a white arrow. Scale bars: 100 μm in (A and B) and 50 μm in respective insets; 200 μm in (C–F) and 100 μm in respective insets; 200 μm in (G, I, K, and M); 50 μm in (Η, J, L, and N); and 25 μm in (O and P) and respective insets. See also Figure S1.

**Figure 2:**
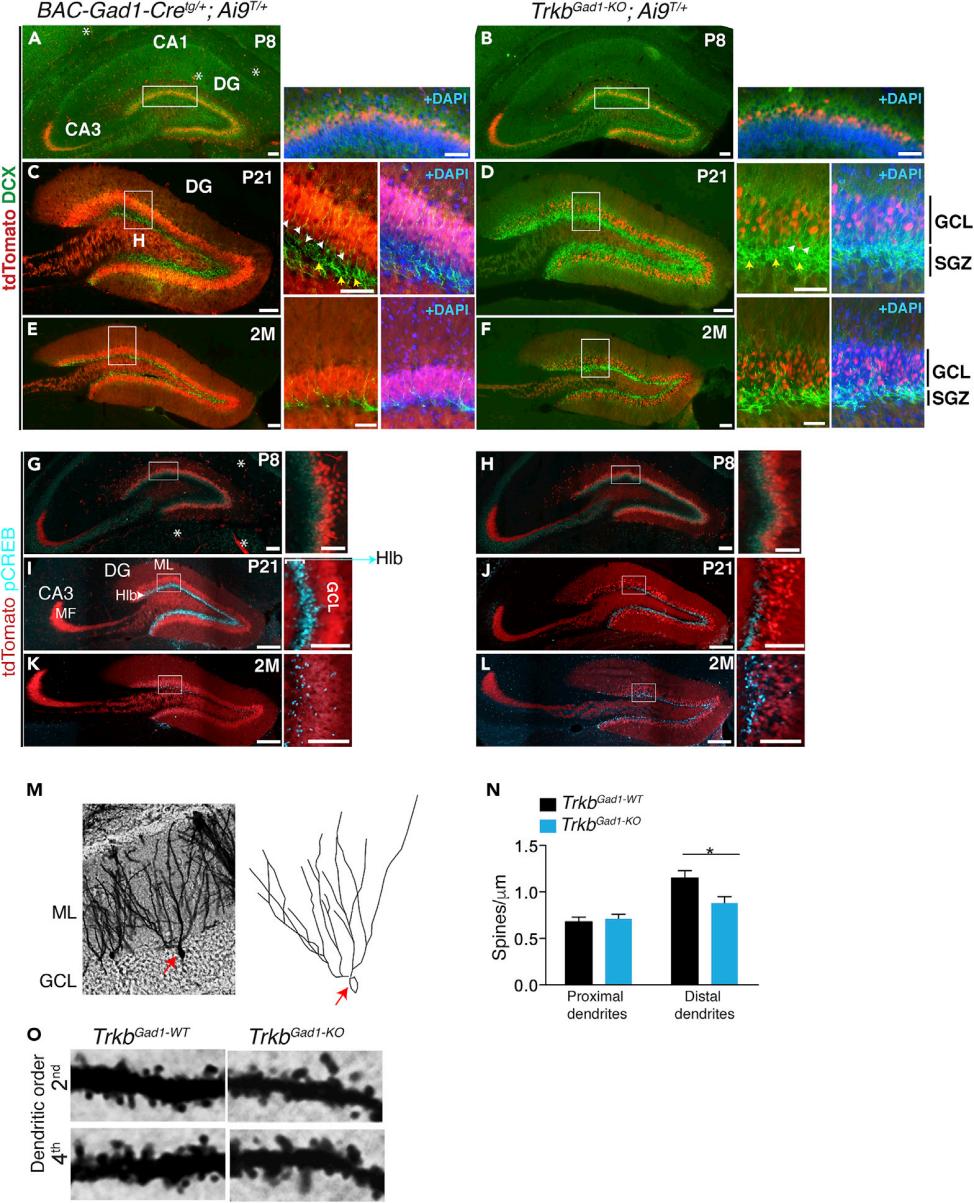
TrkB Signaling Regulates CREB Activation in Immature DGCs and the Integration and Maturation of These Cells into the GCL (A**–**F) Immature DGCs accumulate at the hilar border in the absence of TrkB signaling. (A, C, and E) and (B, D, and F) Representative images of hippocampal coronal sections from controls (*BAC-Gad1-Cre*^*tg/+*^; *Ai9*^*T/+*^) and mutants (*Trkb*^*Gad1-KO*^; *Ai9*^*T/+*^), respectively, at P8, P21, and 2M immunostained for doublecortin (DCX) (green) and superimposed to tdTomato signal (red). At P8, DCX immunostaining shows mainly colocalization with endogenous tdTomato (red) indicating migrating DGCs and a similar pattern between controls and mutants (A and B and insets). DAPI staining (blue). At P21, however, DCX immunostaining shows accumulation of immature DGCs in the subgranular zone (SGZ) of mutants compared with controls (C and D and respective insets). At 2M, as expected, very few DCX-positive DGCs are present in the SGZ of controls (E and insets), whereas in mutants the SGZ appears still abundant in DCX-positive cells, although reduced compared with P21 (F and insets). Scale bars: 100 μm in (A and B) and respective insets; 100 μm in (C–F) and 50 μm in respective insets. White arrowheads indicate examples of colocalization between tdTomato and DCX occurring only in immature neuron DGCs beginning to migrate and mature in the GCL, but not in neural precursors (yellow arrows). (G–L) Representative images of hippocampal coronal sections and insets from controls (G, I, and K, *BAC-Gad1-Cre*^*tg/+*^; *Ai9*^*T/+*^) and mutants (H, J, and L, *Trkb*^*Gad1-KO*^; *Ai9*^*T/+*^), respectively, at P8, P21, and 2M immunostained for phosphoCREB (pCREB). The time course analysis shows pCREB peak of activation at P21 in control mice appearing in the lower third of the granule cell layer (GCL) at the hilar border (Hlb) where newborn immature neurons reside (I and inset). pCREB immunoreactivity declined by 2M age (K and inset). *Trkb* deletion in immature DGCs induces reduced activation of CREB at P21 compared with controls (J and I) followed by a decline at 2M (L). Scale bars: 100 μm in (G and H) and 50 μm in insets; 200 μm in (I–L) and 100 μm in insets. MF, mossy fibers; ∗examples of autofluorescence in structures like blood vessels. (M–O) Golgi staining of newly formed DGCs to analyze dendritic density in the presence and absence of TrkB signaling. (M) Representative image of 2M-old Golgi-stained granule cells in the GCL and drawing of the indicated (arrow) cell showing branching ramification. (N) A similar number of dendritic spines was found between genotypes in proximal dendrites (second to third order, *Trkb*^*Gad−1-WT*^, 0.68 ± 0.05; n = 33 cells from 4 mice; *Trkb*^*Gad−1-KO*^, 0.71 ± 0.05; n = 34 cells from 3 mice; p = 0.99) of DGCs, whereas a significant reduction in dendritic spines was observed along distal dendrites in mutant mice compared with control littermate (fourth order, *Trkb*^*Gad1-WT*^, 1.15 ± 0.08; n = 5 cells from 4 mice; *Trkb*^*Gad1-KO*^, 0.88 ± 0.07; n = 19 cells from 3 mice; ∗p = 0.02). Data are number of spines per 1-μm dendritic segment ± SEM. (O) Representative images of 10-μm dendritic segment of second and fourth order from mutants and control mice. See also Figure S2.

To determine the effect of *Trkb* deletion in immature DGCs, we first performed a time course analysis of the DG development (P8, P21, and 2 months [2M]). Thus, the Ai9 reporter line was crossed to the *Trkb*^*Gad1-KO*^ strain to be able to follow recombined cells. At P8 the forming GCL in the *Trkb*^*Gad1-KO*^; Ai9^T/+^ mice was comparable with controls, whereas at P21, and in the adult stage, fewer tdTomato-positive cells were integrated into the GCL of mutants compared with controls (Figures 1A–1F and insets). These data were corroborated by Calbindin staining, a marker of DGCs' maturity, showing a similar pattern (Figures 1G–1N). However, a cell death marker caspase 3 (Porter and Janicke, 1999), for example, at P21, when the phenotype in the GCL is apparent, did not show increased apoptosis in mutants compared with control mice (Figures 1O and 1P). This was consistent with DAPI staining highlighting cell nuclei and a similar thickness of the GCL between mutants and controls at all ages analyzed (Figures 1A–1F, and respective insets). The data suggested a delay in the migration and integration time of newly formed DGCs in the GCL rather than reduced cell survival. The presence of the forming MF bundles and their terminals targeting the hippocampal CA3 region as revealed by both tdTomato fluorescent signal (Figures 1A–1F) and Calbindin immunostaining (Figures 1G, 1I, 1K, and 1M) further supported this notion. Therefore, we performed a time course analysis for DCX immunostaining to specifically analyze immature DGCs beginning to migrate and integrate into the GCL. Although less evident at P8, at P21 there was an increase in DCX-positive (DCX+) cells in the SGZ of mutant compared with control mice (Figures 2A–2D, and respective insets). In control sections DCX immunofluorescence showed the typical progressive decrease with age (Figures 2C and 2E and insets), whereas in mutants there appeared to be a delay in the progression of immature DGCs into GCL supported by visibly less accumulated DCX+ cells in the SGZ and more tdTomato+ cells in the GCL at 2M (Figures 2D and 2F and insets). However, mutants at 2M still showed accumulation of DCX+ cells compared with controls (Figures 2D–2F and insets). These data were also supported by Ki67 immunostaining, a cell proliferation marker (Scholzen and Gerdes, 2000), showing similar proliferation of progenitors at different stages between mutants and controls (Figures S2A–S2F). Next, we asked how absence of TrkB signaling impairs integration of immature DGCs into the forming GCL. CREB signaling plays a key role in adult hippocampal neurogenesis by regulating the development and survival of newly generated immature DGCs downstream of GABA-mediated excitation (Jagasia et al., 2009). GABAergic development requires BDNF/TrkB signaling, as shown, for example, in the development of inhibition in the cortex (Hong et al., 2008). Therefore, we performed a time course analysis of CREB activation in the DG of *Trkb*^*Gad1-KO*^; Ai9^T/+^ mutant and control mice at age P8, P21, and 2M. This analysis revealed a peak of activation of CREB, measured through its phosphorylation (pCREB), at P21 in control mice. As reported previously for the adult DG (Jagasia et al., 2009), pCREB was predominantly detected in the lower third of the DGL at the hilar border where newborn immature neurons reside (Figures 2G and 2I and insets). pCREB immunoreactivity declined by 2M age (Figure 2K and inset) coinciding with reduced neurogenesis. In the absence of TrkB signaling, however, there was reduced activation of CREB at P21 compared with controls followed by a decline at 2M (Figures 2H, 2J, and 2L). However, CREB activation at 2M was apparently higher in mutants than controls, reflecting the accumulation of DCX-positive cells at this age (Figures 2C–2F). To assess the maturation of the newly formed DGCs, we analyzed dendritic spine density of Golgi-stained granule cell in 2M-old mutant and control mice. The number of dendritic spines was similar between genotypes in proximal dendrites, whereas there was a significant reduction of dendritic spines along distal dendrites in *Trkb*^*Gad1-KO*^ mice compared with control *Trkb*^*Gad1-WT*^ (Figures 2M–2O). Thus, absence of TrkB signaling in immature DGCs reduces CREB activation, possibly through affected early GABA action, and delays the integration and maturation of these cells into the GCL.

### Deletion of *Trkb* from Immature DGCs Alters Coherent Network Oscillations and GABAergic Signaling in Developing CA3 Neurons

**Figure 3:**
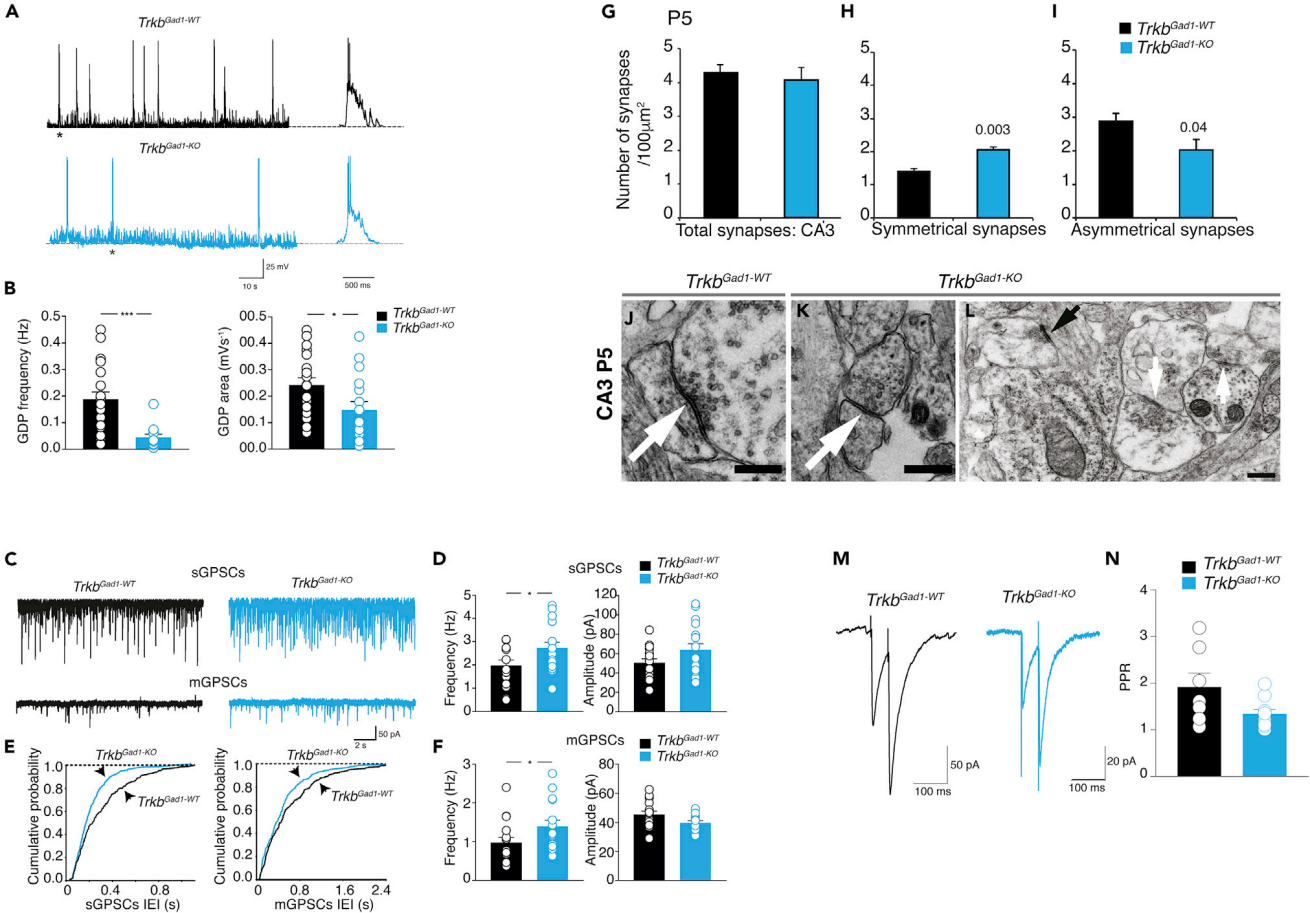
Reduced Expression of Coherent Network Oscillations in CA3 Hippocampal Neurons of *Trkb*^*Gad1-KO*^ Mice (A) GDPs recorded from P3 to P9 *Trkb*^*Gad1-WT*^ and *Trkb*^*Gad1-KO*^ mice. An expanded timescale for the GDPs marked with an asterisk is shown on the right. (B) Summary plots showing the mean frequency and the mean area of GDPs obtained from *Trkb*^*Gad1-WT*^ and *Trkb*^*Gad1-KO*^ mice; GDPs (frequency: control 0.019 ± 0.003 Hz [n = 20 from 6 pups], *Trkb*^*Gad1-KO*^ 0.004 ± 0.001Hz [n = 14 from 3 pups], ∗∗∗p < 0.0001); area (control 0.024 ± 0.003 mVs^−1^, *Trkb*^*Gad1-KO*^ 0.015 ± 0.003 mVs^−1^, ∗p = 0.03). (C) Spontaneous and miniature GPSCs recorded from CA3 principal cells in hippocampal slices from P3 to P9 in *Trkb*^*Gad1-WT*^ and *Trkb*^*Gad1-KO*^ mice. (D) Summary plots showing the mean frequency and amplitude of sGPSCs obtained from *Trkb*^*Gad1-WT*^ (n = 15 from 3 pups) and *Trkb*^*Gad1-KO*^ (n = 18 from 6 pups) mice, respectively (sGPCSs frequency: *Trkb*^*Gad1-WT*^ 1.97 ± 0.2 Hz, *Trkb*^*Gad1-KO*^ 2.74 ± 0.2 Hz, ∗p = 0.03; sGPCSs amplitude: *Trkb*^*Gad1-WT*^ 50.3 ± 4 pA, *Trkb*^*Gad1-KO*^ 64 ± 6 pA, p = 0.09). (E) Cumulative distributions of interevent interval (IEI) of sGPSCs and mGPSCs for cells shown in (C). (F) Summary plots showing the mean frequency and amplitude of mGPSCs obtained from *Trkb*^*Gad1-WT*^ (n = 16 from 5 pups) and *Trkb*^*Gad1-KO*^ (n = 15 from 4 pups) mice, respectively (mGPSCs frequency: *Trkb*^*Gad1-WT*^ 0.97 ± 0.1 Hz, *Trkb*^*Gad1-KO*^ 1.39 ± 0.2 Hz, ∗p = 0.04; mGPSCs amplitude: *Trkb*^*Gad1-WT*^ 45.5 ± 2.2 pA, *Trkb*^*Gad1-KO*^ 39.5 ± 1.7 pA, p = 0.07). Values are mean ± SEM, p statistic from unpaired Student's t tests. (G–L) Increased number of GABAergic synapses in the CA3 region of *Trkb*^*Gad1-KO*^ mice. Number of synapses in the CA3 hippocampal region/100 μm^2^ at P5, *Trkb*^*Gad1-WT*^ (n = 4 mice), *Trkb*^Gad1−KO^ (n = 3 mice). (G) Total, *Trkb*^*Gad1-WT*^, 4.28 ± 0.25, *Trkb*^*Gad1-KO*^, 4.07 ± 0.38, p > 0.05. (H) Symmetrical synapses, *Trkb*^*Gad1-WT*^, 1.40 ± 0.09, *Trkb*^*Gad1-KO*^, 2.06 ± 0.08, p = 0.003. (I) Asymmetrical synapses, control, 2.88 ± 0.25, *Trkb*^*Gad1-KO*^, 2.01 ± 0.32, p = 0.04. (J and L) Representative electron micrographs of single symmetrical synapses (white arrows) from *Trkb*^*Gad1-WT*^ (J) and *Trkb*^*Gad1-KO*^ (K) mice at P5. (L) Representative electron micrograph showing one asymmetrical synapse (black arrow) and two neighboring symmetrical synapses (white arrows) in the CA3 region of a *Trkb*^*Gad1-KO*^ mouse at P5. Note the presence of a clear postsynaptic density at the asymmetric synapse that is lacking from the symmetric synapses. Values are mean ± SEM, p statistic from unpaired one-tailed Student's t tests. Scale bars: 0.5 μm in (J and K) and 500 μm in (L). (M and N) No significant changes in the probability of GABA release at MF-CA3 synapses of *Trkb*^*Gad1-KO*^ mice. (M) Representative examples of synaptic currents evoked in CA3 principal cells by pair stimulation of MF (50-ms interval). (N) Summary plots showing the mean paired-pulse ratio (PPR) values obtained in *Trkb*^*Gad1-WT*^ (n = 7 from 5 pups), 1.91 ± 0.3, and in *Trkb*^*Gad1-KO*^ mice (n = 10 from 9 pups), 1.34 ± 0.1; p = 0.1. See also Figures S3 and S4.

We then asked what impact the absence of TrkB signaling in the immature DGCs would have on the establishment of hippocampal neural circuits. It is well known that the CA3 pyramidal cells and GABAergic interneurons in the hilus are the primary targets of MFs, the axons of DGCs that convey information from the entorhinal cortex (EC) to the hippocampus proper (Acsady et al., 1998). Moreover, although GDPs can be recorded from the entire hippocampus, their occurrence in the CA3 region is facilitated by the extensive network of excitatory collaterals and by the presence of intrinsic bursts that can drive other neurons to fire (Safiulina et al., 2008). Early in postnatal development, DGCs exhibit a mixed GABAergic/glutamatergic phenotype, and in addition to glutamate, MF can also release GABA (Safiulina et al., 2006). Thus, we first assessed the effect of *Trkb* loss in immature DGCs on coherent network oscillations in the developing CA3 area of the hippocampus. GDPs were recorded in current clamp conditions early in postnatal life (P3–P9) from *Trkb*^*Gad1-KO*^ mice and controls. As expected (Ben-Ari et al., 2012), GDPs were characterized by recurrent GDPs with superimposed fast action potentials, separated by silent periods (Figure 3A). We observed a significant decrease in GDP frequency and the underlying area in *Trkb*^*Gad1-KO*^ mice compared with control littermates (Figure 3B). Several factors may contribute to the observed GDP disruption such as altered neuronal excitability in CA3 pyramidal neurons or early inhibitory action of GABA at the network level. To verify whether changes in cell excitability may contribute to GDP alterations, we measured spike frequency in response to depolarizing current pulses of increasing intensities, delivered at −60 mV membrane potential in the presence of GABA_A_ and AMPA/NMDA receptors blockers. No differences in firing threshold and frequencies were observed between the two genotypes (Figures S3A and S3B), suggesting that changes in neuronal excitability were not involved in GDP disruption. GDPs are generated within a local network by the interplay of GABA and glutamate, both depolarizing and excitatory (Ben-Ari et al., 2012). Thus, their impairment may also reflect alterations either in GABAergic or glutamatergic signaling. We recorded spontaneously, pharmacologically isolated GABA_A_-mediated postsynaptic currents (spontaneous GABA_A_-mediated postsynaptic currents [sGPSCs], in the presence of DNQX, 20 μM, and DL-APV, 100 μM) and AMPA-mediated postsynaptic currents (spontaneous excitatory postsynaptic currents [sEPSCs], in the presence of DL-APV, 100 μM, and bicuculline, 10 μM) from P3 to P9 CA3 principal cells in both genotypes. *Trkb*^*Gad1-KO*^ mice exhibited a significant increase in frequency, but not in amplitude, of sGPSCs compared with controls (Figures 3C and 3D). We obtained similar results for miniature events (mGPSCs) recorded in the presence of tetrodotoxin (TTX, 1 μM) (Figure 3C,3E, 3F). No significant changes in frequency and amplitude of spontaneous and miniature glutamatergic events (sEPSCs and mEPSCs) were detected between control and *Trkb*^*Gad1-KO*^ mice (Figures S4A–S4F). These results indicate that specifically perturbing presynaptic BDNF/TrkB signaling in immature DGCs at early postnatal age selectively affects GABAergic but not glutamatergic transmission in the downstream CA3 hippocampal area. Thus the altered GABAergic signaling may contribute to the disruption of GDPs.

### Increased GABAergic Synapses in CA3 Area of *Trkb*^*Gad1-KO*^ Mice

The enhanced frequency of spontaneous and miniature GPSCs observed in *Trkb*^*Gad1-KO*^ mice may depend on an increased number of release sites and/or an increase in the probability of GABA release. We used electron microscopy (EM) and electrophysiology to measure the number of release sites and the probability of GABA release, respectively. In EM experiments we determined the number of “symmetric” (GABAergic) versus “asymmetric” (glutamatergic) synapses in the CA3 hippocampal area of *Trkb*^*Gad1-KO*^ and control mice at P5 and young adult age. The total number of synapses was not significantly different between the two genotypes at P5 and young adult age (Figures 3G and S4G), whereas we found a significant increase in the number of symmetric synapses in *Trkb*^*Gad1-KO*^ mice compared with controls both at P5 and young adult age (Figures 3H and S4H). Besides, we observed a statistically significant decrease in the number of asymmetric synapses in *Trkb*^*Gad1-KO*^ mice at P5 compared with control mice (Figure 3I). The reduced number of asymmetric synapses was not associated with a reduction of spontaneous EPSCs (Figure S4A) probably because when compared with GABAergic ones, at this developmental stage glutamatergic synapses may not be all functional (Durand et al., 1996). Conversely, in adult mice, the increase in symmetric synapses in mutants was not accompanied by a significant change in the number of asymmetric synapses (Figure S4I). No obvious gross morphological differences in the appearance of symmetric synapses between the two genotypes were noticed (Figures 3J–3L). In electrophysiological experiments, we determined the probability of GABA release from MF terminals measuring the paired-pulse ratio (PPR) among two pulses delivered to DGCs at 50-ms interval (in the presence of DNQX and DL-AP5 to block AMPA and NMDA receptors, respectively). In agreement with their MF origin (Safiulina et al., 2006), synaptic currents exhibited a strong paired-pulse facilitation and were significantly reduced by the mGluR agonist L-AP4 both in controls (43.3% ± 6.3%, n = 6; p = 0.03) and *Trkb*^*Gad1-KO*^ mice (56.3% ± 6.0%, n = 8; p = 0.008, respectively; Wilcoxon test). We found similar values of PPR in both genotypes (Figures 3M and 3N) suggesting that changes in the probability of GABA release from DGCs do not contribute to the observed effects. Overall, these experiments revealed that the increase in GABAergic synapses is the primary determinant of the enhanced frequency of spontaneous and miniature GPSCs and are supportive of a presynaptic role of DGCs/TrkB signaling in the establishment of early synchronized activity and a proper excitatory/inhibitory balance within the CA3 hippocampal area.

### Altered Chloride Homeostasis and the Polarity of GABA Action in Targeted Neurons

**Figure 4:**
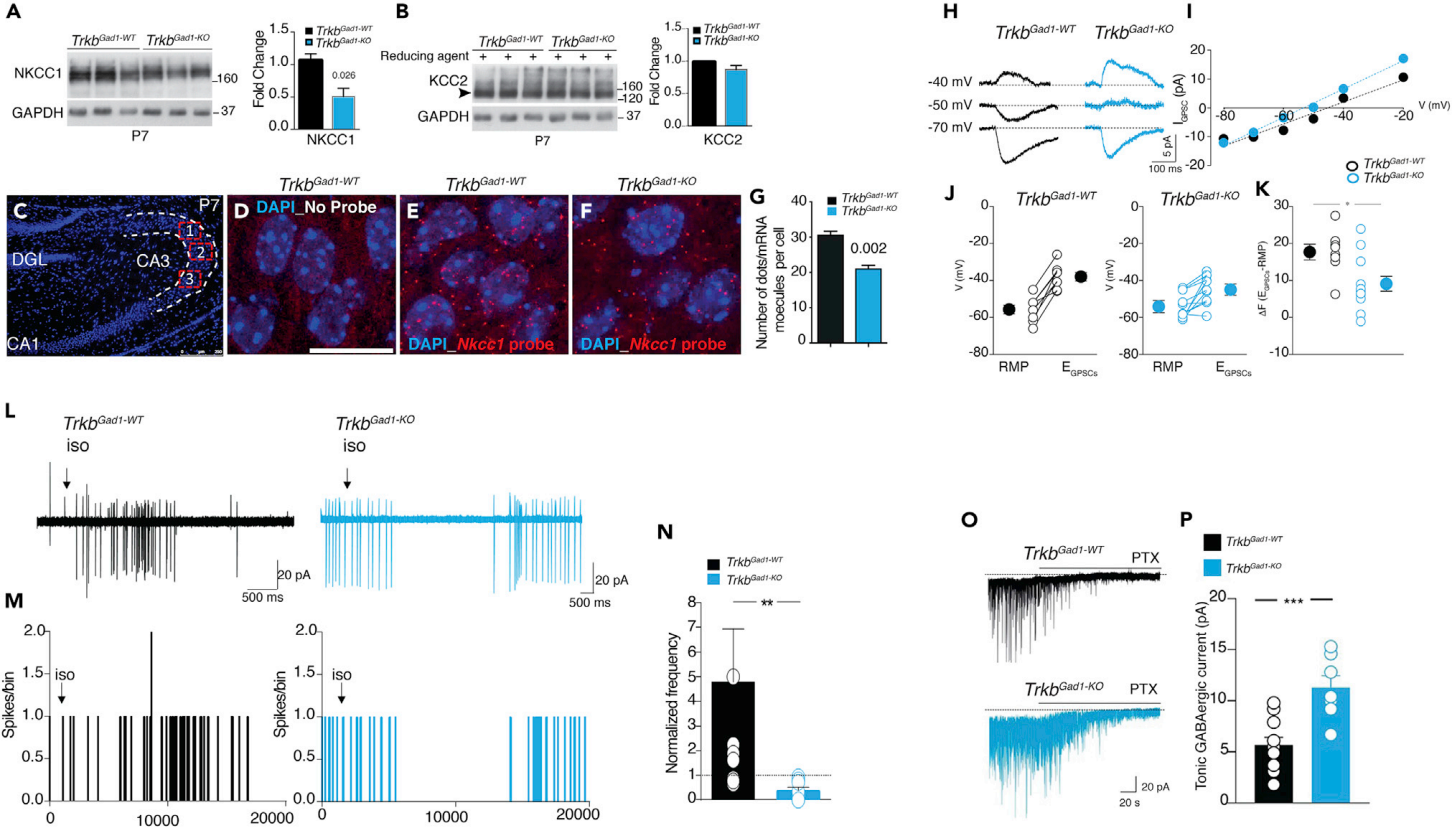
Reduced Expression of *Nkcc1* and Altered Direction of GABA Action at Immature MF-CA3 Synapses in *Trkb*^*Gad1-KO*^ Mice (A and B) Representative western blots from P7 hippocampal lysates and relative quantification of NKCC1 protein levels (P7, *Trkb*^*Gad1-WT*^ [n = 6], 1.065 ± 0.1013; *Trkb*^*Gad1-KO*^ [n = 6], 0.5086 ± 0.1261, p = 0.026) (A) and KCC2 monomer levels (P7, *Trkb*^*Gad1-WT*^ [n = 6], 0.9846 ± 0.01889, *Trkb*^*Gad1-KO*^ [n = 6], 0.8694 ± 0.06086, p = 0.1) (B). GAPDH, loading control. (C–G) Single-molecule fluorescence *in situ* hybridization (smFISH) was used to detect and count individual *Nkcc1* RNA molecules in single cells of the CA3 region at P7. (C) Representative image of a P7 hippocampal section stained with DAPI. Highlighted are three random fields imaged in the CA3 region for quantification of single-molecule RNA (more details in Transparent Methods). (D–F) Representative images from the CA3 regions of control and mutant mice highlighting the single cells by DAPI nuclear staining. The red spots corresponding to single mRNA molecules derived from the transcription of *Nkcc1* are detected with the Quasar570 fluorophore-labeled oligonucleotide probe library in single cells of the CA3 hippocampal region (probe details in Table S4) (E and F); no probe control (D). (G) Quantification of single mRNA molecules per cell (*Trkb*^*Gad1-WT*^, 30.67 ± 0.9943 from n = 376 cells; *Trkb*^*Gad1-KO*^, 21.16 ± 0.8630 from n = 396 cells, p = 0.002; n = 3 P7 pups each genotype). DGL, dentate granule layer. Scale bars: 250 μm in (C) and 50 μm in (D–F). (H–K) Reduced driving force for GABA-mediated postsynaptic currents (GPSCs) at MF-CA3 synapses in *Trkb*^*Gad1-KO*^ mice. (H) Representative traces of GPSCs evoked at three different holding potentials in CA3 principal cells by MF stimulation (gramicidin-perforated patches) in *Trkb*^*Gad1-WT*^ and *Trkb*^*Gad1-KO*^ mice. (I) Amplitudes of GPSCs (I_GPSC_) shown in (H) are plotted against holding potentials (V). (J) Individual RMPs and *E*_GPSCs_ values in CA3 principal cells from control (*n* = 8 from 5 pups) and *Trkb*^*Gad1-KO*^ (*n* = 11 from 7 pups). Larger symbols on the left and right refer to mean ± SEM values (RMPs, *Trkb*^*Gad1-WT*^, −55.6 ± 2.4 mV; *Trkb*^*Gad1-KO*^, −54.2 ± 2 mV; *Trkb*^*Gad1-WT*^*E*_GPSCs_, −37.9 ± 2.3mV; *Trkb*^*Gad1-KO*^, −44.9 ± 2.3mV). (K) Plot of the driving force (ΔF) for GABA (*ΔF=E*_GPSCs_–*RMP*) in individual experiments from *Trkb*^*Gad1-WT*^ and *Trkb*^*Gad1-KO*^ mice. Larger symbols are mean ± SEM values. *ΔF=* 17.7 ± 2.1mV in *Trkb*^*Gad1-WT*^ and 9.1 ± 2.2mV *in Trkb*^*Gad1-KO*^ mice; ∗p = 0.03, Wilcoxon test. (L–N) Altered GABAergic signaling accounts for network dysfunction in *Trkb*^*Gad1-KO*^ immature hippocampus. Effects of isoguvacine on spontaneous firing of CA3 principal cells in *Trkb*^*Gad1-WT*^ and *Trkb*^*Gad1-KO*^ mice. (L) Two representative examples of changes in spontaneous firing induced by pressure application of isoguvacine (100 μM for 1 s; arrows) to CA3 principal cells (recorded in cell-attached) in *Trkb*^*Gad1-WT*^ (black) and *Trkb*^*Gad1-KO*^ mice (cyan). (M) Interspike interval histograms (bin: 10 ms) for cells shown in (L). (N) Summary plot showing isoguvacine-induced changes in spike frequency (30 s after drug application) normalized to baseline values (30 s before drug application); *Trkb*^*Gad1-WT*^, 4.78 ± 2.1 (n = 9 from 4 pups), *Trkb*^*Gad1-KO*^, 0.39 ± 0.1 (n = 8 from 5 pups); ∗∗p = 0.025. Values are mean ± SEM, p statistic from unpaired Student's t test. (O and P) Increased GABA_A_-mediated tonic conductance in *Trkb*^*Gad1-KO*^ mice. (O) Representative traces of spontaneous GABA_A_-mediated synaptic currents (sGPSCs) recorded from CA3 principal cells before and during application of picrotoxin (PTX, 100 μM bars above the traces) in hippocampal slices obtained from control (n = 11) and *Trkb*^*Gad1-KO*^ mice (n = 7). Note the upward shift of the baseline current and disappearance of sGPSCs after application of PTX (100 μM) in the presence of DNQX (20 μM) and DL-APV (100 μM). (P) Each column represents the mean tonic GABA_A_-mediated conductance measured in controls, 5.7 ± 0.75 pA (n = 11) and *Trkb*^*Gad1-KO*^ mice, 11.3 ± 1.2 pA (n = 7). ∗∗∗p = 0.0008, Mann-Whitney test. See also Figures S5 and Table S4.

The enhanced GABAergic drive to CA3 principal cells may contribute to disrupting coherent network oscillations via a shunting inhibition caused by the premature shift of GABA action from the depolarizing to the hyperpolarizing direction. Immediately after birth, MFs release GABA that exerts a depolarizing and excitatory action on their targets (Sivakumaran et al., 2009). This action depends on the delayed temporal expression of the Cl^−^ exporter KCC2 compared with an initial increased expression of the cation/Cl^−^ importer NKCC1 (Blaesse et al., 2009). Therefore, to assess whether *Trkb* deletion from immature DGCs affects chloride homeostasis, thus altering the polarity of GABA action, we first measured the protein expression levels of NKCC1 and KCC2 by western blot (WB) from P7 hippocampal lysates. We found a significant decrease of NKCC1 in *Trkb*^*Gad1-KO*^ mice compared with controls (Figure 4A). This decrease was still apparent at P50 (Figure S5A). We detected no changes in KCC2 levels of monomeric or oligomeric forms between the two genotypes at P7 (Figure 4B), or at a later age (P50) (Figures S5B and S5C). Next, to visualize, localize, and quantify more precisely the amount of NKCC1 in cells of the CA3 hippocampal region we used single-molecule fluorescence *in situ* hybridization (smFISH) for *Nkcc1* mRNA detection and quantification at P7. As shown in Figures 4C–4G, the number of single mRNA molecules per cell detected in *Trkb*^*Gad1-KO*^ mice was significantly diminished compared with controls, confirming the WB results.

We then tested whether the reduced expression of *Nkcc1* alters the direction of GABA action at immature MF-CA3 synapses. First, we measured the equilibrium potential of MF-evoked GPSCs (E_GPSC_) in CA3 principal cells using gramicidin perforated-patch recordings to prevent changes in intracellular chloride concentration [Cl^−^]_i._ We found that in *Trkb*^*Gad1-KO*^ mice, compared with controls, the equilibrium potential of GABAergic postsynaptic currents (E_GPCs_) evoked by MF stimulation was shifted toward more hyperpolarized potentials (Figures 4H–4K). It is worth noting that whereas in control animals, seven of eight neurons exhibited E_GABA_ values at least 15 mV positive with respect to their resting membrane potentials (RMP), in *Trkb*^*Gad1-KO*^ mice only three of eleven cells exhibited similar values. In contrast, the remaining cells showed only small differences of few millivolts around their RMPs. The RMP values, estimated at the end of the experiments by breaking the membrane, were used to measure the driving force for MF-mediated GPSCs (ΔF_GABA_), being ΔF_GABA_ = E_GPCs_-RMP. These values were significantly different between controls and *Trkb*^*Gad1-KO*^ mice (Figure 4K). Altogether these data suggest that the reduced expression of *Nkcc1* in *Trkb*^*Gad1-KO*^ mice is sufficient to shift E_GABA_ toward more hyperpolarized values, close to their RMPs, thus exerting a shunting inhibition at the network level.

To further assess whether altered GABAergic signaling may account for network dysfunction in *Trkb*^*Gad1-KO*^ immature hippocampus (P3–P9), we used cell-attached recording and examined the effects of isoguvacine, a specific GABA_A_ agonist, on the spontaneous firing of CA3 principal cells. As expected, isoguvacine, applied by pressure from a pipette positioned close to recorded neurons, increased the firing rate of CA3 pyramidal cells in control animals (Khazipov et al., 2004), but it consistently reduced it in *Trkb*^*Gad1-KO*^ mice (Figures 4L–4N). In conclusion, these experiments demonstrate that reduced TrkB signaling in DGCs, early in postnatal development, leads to a reduced expression of the cation-chloride importer NKCC1. The lower [Cl^−^]_i_ produces a premature shift of GABA from the depolarizing to the hyperpolarizing direction with consequent inhibitory effect at the network level.

### Enhanced GABA_A_-Mediated Tonic Inhibition in the Hippocampus of *Trkb*^*Gad1-KO*^ Mice

The data reported in the previous section clearly show that GABA exerts an inhibitory effect on network-driven GDPs via a shunting inhibition. This inhibitory action may be further boosted by a tonic GABA_A_-mediated conductance, following activation of extrasynaptic GABA_A_ receptors by spillover of GABA from adjacent synapses. Such conductance is known to be altered in several forms of neurodevelopmental disorders (Brickley and Mody, 2012, Cellot et al., 2016). To test this hypothesis, we measured the shift in the holding current induced by bath application of the GABA_A_ receptor channel blocker picrotoxin (100μM) in the presence of DNQX (20μM) and DL-APV (100μM). This caused a more pronounced shift in the holding current in *Trkb*^*Gad1-KO*^ compared with control mice (Figures 4O and P). These results suggest that indeed an increased GABA_A_-mediated tonic conductance activated by GABA released from GABAergic interneurons may contribute to altering GDPs.

### The Premature Shift of GABA from the Depolarizing to the Hyperpolarizing Direction Affects the Maturation of CA3 Principal Cells and Impairs LTP at MF-CA3 Synapses

**Figure 5:**
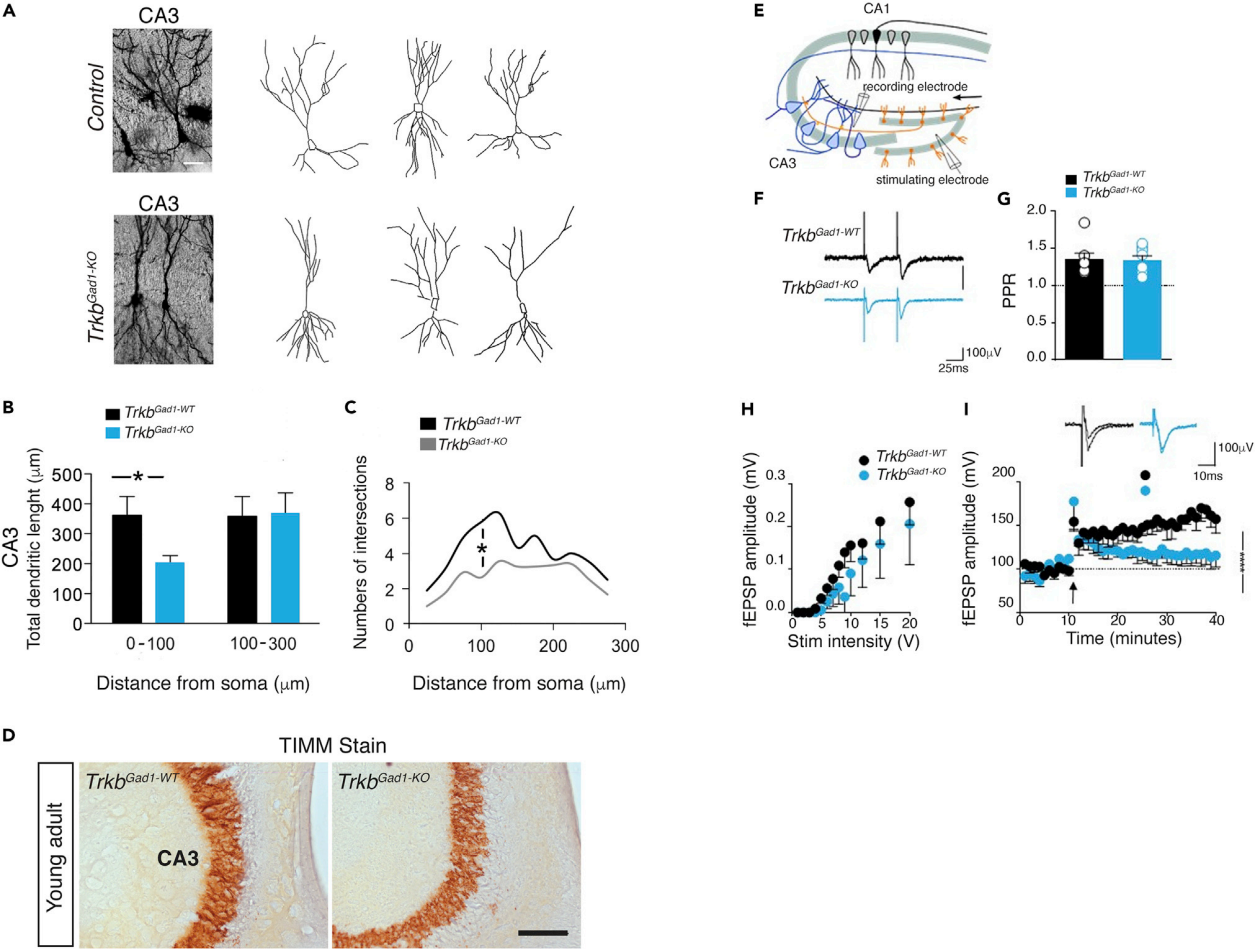
Morphological Alterations of CA3 Principal Cells and Impaired Hippocampal LTP at MF-CA3 Synapses in Adult *Trkb*^*Gad1-KO*^ Mice (A) Representative images of Golgi-stained (left) and neurolucida tracings (right) of CA3 principal cells from young adult control (*Trkb*^*Gad1-WT*^) and *Trkb*^*Gad1-KO*^ mice. (B) Quantitative analysis of dendritic length of CA3 principal neurons in *Trkb*^*Gad1-WT*^ and in *Trkb*^*Gad1-KO*^ mice. Total dendritic length, proximal 1–100 μm from soma (*Trkb*^*Gad1-WT*^, 362.32 ± 65.51; *Trkb*^*Gad1-KO*^, 203.75 ± 23.78, Student's t test: t_(19)_ = 2.59; ∗p = 0.017), and distal 100–300 μm from soma (*Trkb*^*Gad1-WT*^, 358.13 ± 94.33; *Trkb*^*Gad1-KO*^, 369.82 ± 107.61, Student's t test: t_(18)_ = 0.26; p = 0.79). (C) Sholl analysis of principal neurons in *Trkb*^*Gad1-WT*^ and *Trkb*^*Gad1-KO*^ mice. Statistically significant differences across the dendritic trees between genotypes are marked by a vertical line (*Trkb*^*Gad1-WT*^, 5.77 ± 1.0; *Trkb*^*Gad1-KO*^, 2.63 ± 0.4, Student's t test: t_(18)_ = 2.93; ∗p = 0.008); values are mean ± SEM. *Trkb*^*Gad1-WT*^, n = 9 neurons from 4 mice; *Trkb*^*Gad1-KO*^, n = 12 neurons from 3 mice. (D) Representative images of Timm stain per genotype showing similar MF pattern in the CA3 target region of 2M-old mice (n = 3 each group). (E) LTP at MF-CA3 synapses. Cartoon illustrating the experimental paradigm. (F) Sample traces of paired field (f) EPSPs (50-ms interval) evoked in CA3 *stratum radiatum* (*sr*) upon stimulation of MF in *stratum lucidum* (*sl*) in the presence of DL-AP5 (100 μM). (G) Each column represents the mean value of the paired-pulse ratio (PPR, 50-ms interval) obtained from *Trkb*^*Gad1-WT*^ (1.35% ± 9%, n = 7 slices from 4 mice) and from *Trkb*^*Gad1-KO*^ mice (1.33% ± 6%, n = 8 slices from 3 mice), p = 0.92. (H) Input-output relationship of fEPSP responses evoked in CA3 *stratum radiatum* by MFs stimulation in the *stratum lucidum* of *Trkb*^*Gad1-WT*^ (n = 9 slices from 5 mice) and *Trkb*^*Gad1-KO*^ mice (n = 5 slices from 5 mice), Kolmogorov-Smirnov test, p = 0.998. Each point represents the mean ± SEM. (I) The amplitudes of MF-fEPSPs recorded from *Trkb*^*Gad1-WT*^ (n = 4 slices from 3 mice) and *Trkb*^*Gad1-KO*^ mice (n = 5 slices from 4 mice) before and after theta-bursts stimulation (arrow, 10 trains 200 Hz, 50 ms each, repeated twice at 0.1 Hz) of MF was normalized to baseline values and plotted against time. Mean amplitude values of fEPSPs evoked 10 min before and 30 min after LTP induction (30 min, fEPSPs amplitude, *Trkb*^*Gad1-WT*^, 160% ± 2.1%, n = 4 slices from 3 mice; *Trkb*^*Gad1-KO*^, 116.1% ± 0.52%, n = 5 slices from 4 mice, p < 0.001). The insets above the graph show two superimposed average traces of fEPSPs obtained from *Trkb*^*Gad1-WT*^ and *Trkb*^*Gad1-KO*^ mice before and 30 min after LTP induction. Scale bars: 500 μm in (A) and 100 μm in (D).

To investigate whether changes in network activity and GABAergic signaling following *Trkb* deletion from the hippocampal immature DGCs affect CA3 principal cell maturation, we examined the morphology of CA3 principal cells at young adult age (2M) by Golgi staining (Figure 5A). *Trkb*^*Gad1-KO*^ mice showed a significant reduction in proximal dendritic segment length compared with controls (Figure 5B). The number of dendritic intersections was also significantly reduced in *Trkb*^*Gad1-KO*^ mice only on apical dendrites located within 100 μm from the soma. No differences between the two genotypes were detected in more distal dendrites (>100 μm from the soma) (Figure 5C), or in basal dendrites (ANOVA, F_6,114_ = 0.283; p > 0.05). However, the observed morphological alterations of the CA3 principal cells were not due to lack of MF terminals, making synaptic contact onto CA3 principal cells. The use of Timm stain, which detects the Zn2+-containing MF terminals, revealed similar MF bundles between *Trkb*^*Gad1-KO*^ mice and controls (Figure 5D), confirming both the tdTomato reporter signal (Figures 1A–1F) and the Calbindin immunostaining (Figures 1G, 1I, 1K, and 1M).

Hence, we tested the hypothesis that morphological alterations in CA3 principal cells due to the absence of presynaptic TrkB signaling may alter the development of functional neuronal circuits and storage of information leading to altered long-term potentiation (LTP) and behavioral impairment in adult animals. MF-CA3 synapses are known to undergo robust NMDA-independent LTP following theta-burst activation of the MF pathway in *stratum lucidum* (Nicoll and Schmitz, 2005). Therefore, field EPSPs (fEPSPs) evoked by stimulation of MF in *stratum lucidum* were routinely recorded in hippocampal slices (from 2- to 3M-old animals) in the presence of DL-AP5 (100 μM), a selective NMDA receptor antagonist. In basal conditions (0.1-Hz stimulation), we detected no changes in synaptic transmission, cell excitability, or short-term plasticity between the two genotypes (Figures 5E–5H). The PPR, a typical presynaptic form of short-term plasticity, was similar between *Trkb*^*Gad1-KO*^ mice and control littermates (Figures 5F and 5G). However, whereas theta-burst stimulation induced persistent changes in synaptic efficacy in control animals, the same stimulation induced only a transient increase in synaptic efficacy in *Trkb*^*Gad1-KO*^ mice that further decreased over time to baseline levels. There was a significant difference in LTP magnitude between the two genotypes (Figure 5I).

### *In Vivo*, LTP Is Depressed at the Schaffer Collateral-CA1 Synapses in *Trkb*^*Gad1-KO*^ Mice

**Figure 6:**
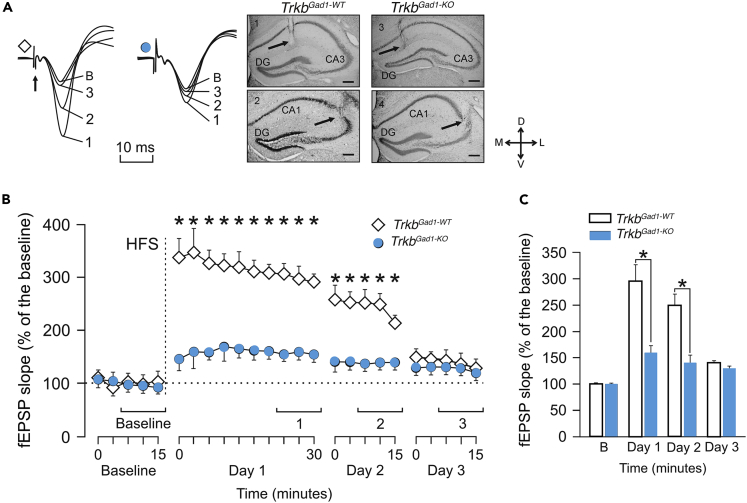
*In Vivo* Recordings of LTP in the CA1 Hippocampal Area Following Electrical Stimulation of Schaffer Collaterals (A) Representative example fEPSP recordings collected from selected animals of each experimental group at the times indicated in (B). In all cases, the smaller fEPSP was collected before the high-frequency stimulation (HFS) (baseline), whereas the larger one was collected 24–30 min after the HFS. On the right, images illustrating the location of recording (1 and 3) and stimulating (2 and 4) sites (arrows) are shown. (B) Time course of changes in fEPSP slopes (mean ± SEM) following HFS stimulation of Schaffer collaterals. Baseline recordings were collected for 15 min at a rate of 3 pulse/min. As indicated in the Methods, we then evoked LTP by HFS protocol consisting of five trains (200 Hz, 100 ms) of pulses at a rate of 1/s. This protocol was presented six times in total, at intervals of 1 min. After HFS, we presented the same single stimulus used to generate baseline records at the initial rate (3/min) for another 30 min. Additional sets of recordings (15 min each) were obtained 24 and 48 h after HFS. fEPSPs are given as a percentage of baseline (100%) values. Following the HFS session, fEPSPs were recorded for 30 min on day 1 and for 15 min on the following days. ∗Significant differences between *Trkb*^*Gad1-WT*^ and *Trkb*^*Gad1-KO*^ group (F_(24,336)_ = 36.839; p < 0.001; n = 8 animals per group. Two-way repeated measures ANOVA, one factor repetition). (C) fEPSP data (mean ± SEM) included in each histogram (baseline, days 1, 2, and 3) were collected for the time intervals indicated in (B). ∗[F_(3,118)_ = 93.67; p < 0.001; n = 8 animals per group. Two-way repeated measures ANOVA, one factor repetition]. Scale bars: 250 μm in (A). Abbreviations: D, L, M, and V, dorsal, lateral, medial, ventral, respectively; DG, dentate gyrus. See also Figure S6 and Tables S1, S2, and S3.

The CA3 pyramidal neurons project mainly to the CA1 pyramidal neurons via Shaffer collaterals. As presynaptic deletion of *Trkb* affected synaptic strength at MF-CA3 synapses, we asked if consequently synaptic plasticity was also altered at Schaffer collateral-CA1 synapses. Therefore, we investigated synaptic plasticity at Schaffer collateral-CA1 synapses where LTP is known to last for hours to days in alert-behaving adult animals. No changes in basal synaptic transmission and short-term plasticity (paired-pulse facilitation) were observed between the two genotypes and additional control groups (*BAC-Gad1-Cre*^*tg/+*^ animals compared with wild-type [WT] littermates) (Figures S6A and S6B, and Table S1). However, LTP evoked in the control littermates (*Trkb*^*Gad1-WT*^) was significantly larger and longer lasting (up to 48 h) compared with that evoked in the mutant *Trkb*^*Gad1-KO*^ group (Figures 6A–6C, and Table S2). Also, the *BAC-Gad1-Cre*^*tg/+*^ animals compared with WT littermates presented similar LTP lasting for 48 h (Figures S6C–S6E, and Table S3). These data suggest that alert-behaving *Trkb*^*Gad1-KO*^ animals are unable to present a significant increase in fEPSP slopes evoked at the CA3-CA1 synapses following the high-frequency stimulation session applied to the Schaffer collaterals/commissural pathway. Therefore, selective *Trkb* deletion in immature DGCs severely alters the establishment of hippocampal circuitry and storage of information downstream of DGCs, leading to synaptic plasticity impairment in adulthood.

### The Behavioral Correlate of Neural Circuits Downstream of MF-CA3 Synapses Is Selectively Affected in *Trkb*^*Gad1-KO*^ Mice

**Figure 7:**
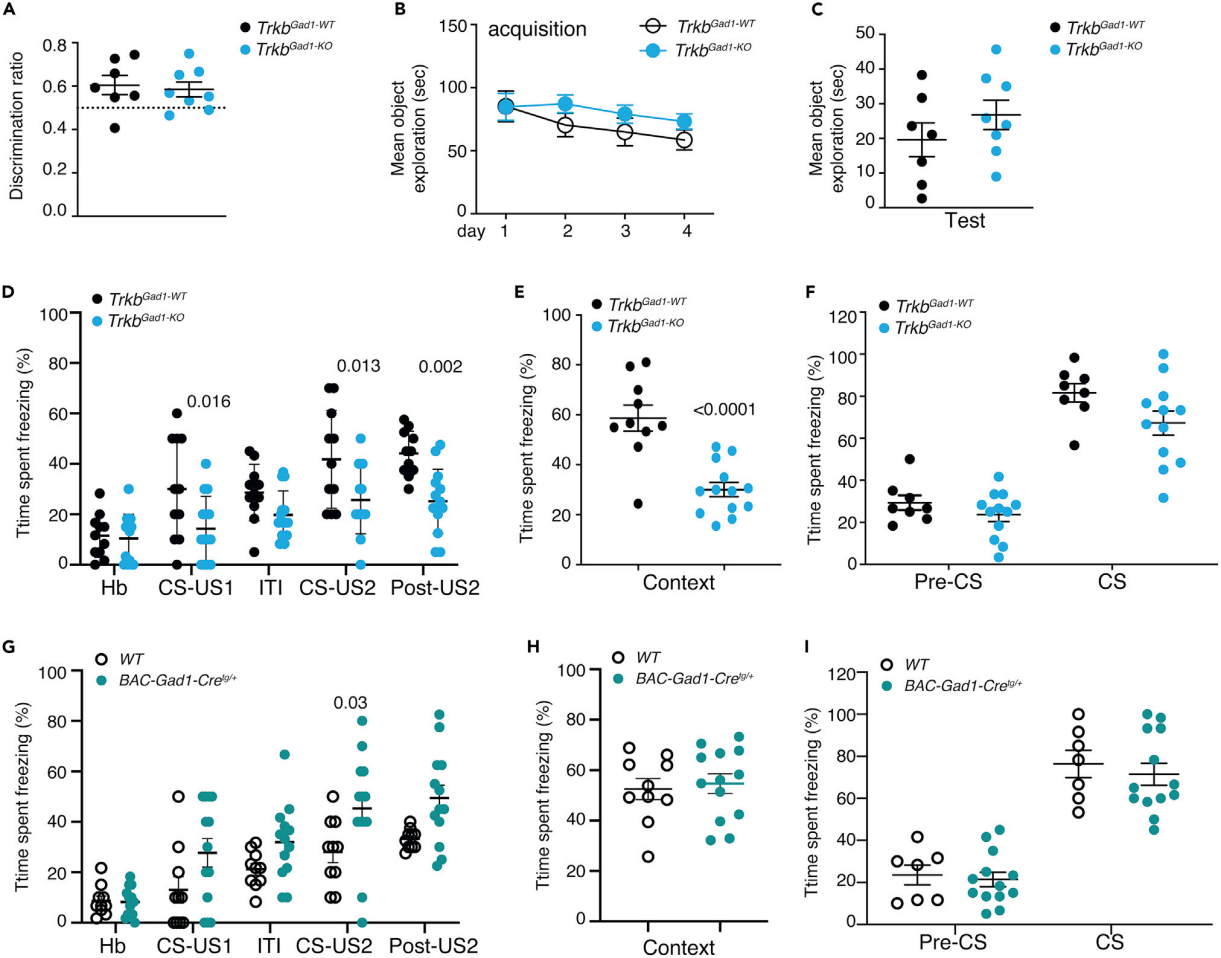
Normal Context-Object Recognition but Affected Neutral Context Recognition in *Trkb*^*Gad1-KO*^ Mice (A–C) Context-object recognition test. (A) Mean discrimination ratio was calculated as a ratio of the time spent exploring an object not previously paired with the context over the total time spent observing both paired and unpaired objects. No difference was found between genotypes (*Trkb*^*Gad1-WT*^ [n = 7] 0.61 ± 0.04; *Trkb*^*Gad1-KO*^ [n = 8] 0.59 ± 0.03, mean ± SEM, unpaired Student's t test, p = 0.7195). (B) Mean total object exploration time during the 4-day acquisition phase was similar between *Trkb* mutant and control mice and decreased significantly over time (two-way ANOVA repeated measures: F_(3,39)_ = 3.276, p = 0.031, main effect of time). (C) The mean total object exploration time analyzed during the test session was also not significantly different between genotypes (*Trkb*^*Gad1-WT*^, 19.61 ± 4.9; *Trkb*^*Gad1-KO*^, 26.78 ± 4.23; mean ± SEM; unpaired Student's t test: t_(13)_ = 1.112, p = 0.2861). (D–F) Fear conditioning test. (D) Acquisition phase. Two-way ANOVA repeated measures (*Trkb*^*Gad1-WT*^, n = 10; *Trkb*^*Gad1-KO*^, n = 13) revealed a main effect of genotype in freezing behavior (F_(1,23)_ = 10.56, p = 0.0035), a main effect of test stage (F_(4,92)_ = 22.1, p < 0.0001), and an interaction between genotype and test stage (F_(4,92)_ = 3.072, p = 0.0201). To explore the observed interaction, Sidak's multiple corrected comparisons showed a statistically significant difference between genotypes in the percentage time spent freezing during the CS-US1 (*Trkb*^*Gad1-WT*^, 30.00% ± 6.03%; *Trkb*^*Gad1-KO*^, 14.29% ± 3.43%; mean ± SEM, p = 0.0166), during the CS-US2 (*Trkb*^*Gad1-WT*^, 41.82% ± 5.85%; *Trkb*^*Gad1-KO*^, 25.71% ± 3.59; p = 0.013), and in the post-US2 (*Trkb*^*Gad1-WT*^, 44.09% ± 2.68%; *Trkb*^*Gad1-KO*^, 25.18% ± 3.39%; p = 0.002). (E) Twenty-four hours after conditioning, mice were first tested for contextual fear conditioning. A statistically significant difference in the time spent freezing was found between control (n = 10), 58.72% ± 5.22%, and *Trkb*^*Gad1-KO*^ (n = 13) mice, 30.04% ± 2.84%; mean ± SEM, unpaired Student's t test, p < 0.0001, two-tailed. (F) Twenty-four hours later mice were tested for cued fear conditioning. Two-way ANOVA repeated measures (*Trkb*^*Gad1-WT*^, n = 8; *Trkb*^*Gad1-KO*^, n = 12) revealed a main effect of test stage (F_(1,18)_ = 92.3, p < 0.0001) and a main effect of genotype (F_(1,18)_ = 5.446, p = 0.0314), but no interaction (F_(1,18)_ = 0.7558, p = 0.3961). Sidak's multiple comparisons test showed no significant difference between control and mutant mice in freezing responses (%) (*Trkb*^*Gad1-WT*^, 81.67% ± 4.40%; *Trkb*^*Gad1-KO*^, 67.22% ± 5.77%, p = 0.07). (G–I) Context and cued fear conditioning are unaffected in *BAC-Gad1-Cre* mice. (G) Percentage of time spent freezing during conditioning. Two-way ANOVA repeated measures (*BAC-Gad1-Cre* mice, n = 13; WT control, n = 10) revealed a main effect of genotype in freezing behavior (F_(1,21)_ = 8.477, p = 0.0083) and a main effect of the stage of the test (F_(4,84)_ = 24.43, p < 0.0001), but no interaction between genotype and test stage (F_(4,84)_ = 1.865, p = 0.1241). Sidak's multiple corrected comparisons showed a statistically significant difference between genotypes in the percentage of time spent freezing during the second tone-foot shock presentations (CS-US2, WT: 28.00% ± 4.16%, *BAC-Gad1-Cre*: 45.38% ± 6.06%, p = 0.0305) compared with the other stages (CS-US1, WT 13.00% ± 5.17%, *BAC-Gad1-Cre*: 27.69% ± 5.68%, p = 0.09; post-US2, WT: 33.25% ± 1.24%, *BAC-Gad1-Cre*: 49.42% ± 5.15%, p = 0.052). (H) Twenty-four hours after conditioning, mice were first tested for contextual fear conditioning, and no difference was found in freezing responses (%) between WT (n = 10) and *BAC-Gad1-Cre* mice (n = 13) (% of time spent freezing, WT: 52.53% ± 4.17%, *BAC-Gad1-Cre*: 54.72% ± 3.92%; unpaired Student's t test, p = 0.697, two tailed). (I) Cued fear conditioning was tested 24 h later. Two-way ANOVA repeated measures revealed a main effect of test stage (F_(1,18)_ = 266.3, p < 0.0001), but no effect of genotype (F_(1,18)_ = 0.2818, p = 0.6020). Freezing responses (%) (WT, n = 7, *BAC-Gad1-Cre*, n = 13) (% of time spent freezing, CS, WT: 76.43% ± 6.48%, *BAC-Gad1-Cre*: 71.54% ± 5.25%, p = 0.76). Values are mean ± SEM. ITI, intertrial interval; Hb, habituation; CS, conditioned stimulus; US, unconditioned stimulus. See also Figures S7.

As deletion of *Trkb* in immature DGCs affected synaptic strength at MF-CA3 and Schaffer collateral-CA1 synapses, we asked whether these alterations would specifically affect the processing of information that depends on intact hippocampal regions. Therefore, we tested *Trkb*^*Gad1-KO*^ and control mice in two behavioral tasks, the object-context recognition (OCR) and the contextual fear conditioning (FC). In rats, the OCR has been shown to critically rely on the lateral entorhinal cortex (LEC), which processes the contextual features of an event (Wilson et al., 2013). Mice were first tested in an open field where both mutants and controls showed habituation to the arena and normal anxiety behavior (Figures S7A–S7C). Then, we tested mice in the OCR task (experimental design explained in Figure S7D). Both genotypes showed no difference in the discrimination ratio (Figure 7A). Similarly, the total object exploration time during the acquisition phase and the test session was not significantly different between genotypes (Figures 7B and 7C), suggesting that the LEC is intact in these mutants. We then tested mice for an associative learning task such as contextual FC. In this form of learning an aversive stimulus (electrical shock) is associated with a specific neutral context, or a neutral stimulus (tone). *Trkb*^*Gad1-KO*^ mice showed impaired neutral context acquisition/recognition but normal response to a conditioned neutral stimulus compared with control littermates or the *BAC-Gad1-Cre* (Figures 7D–7F and 7G–7I). Because contextual FC involves all three hippocampal areas, DG, CA3, and CA1, to integrate impulses from the amygdala (Lee and Kesner, 2004), a structure in the brain needed for the expression of fear, these results further support that presynaptic depletion of TrkB signaling from immature DGCs affects specifically neuronal circuits located within the hippocampus proper downstream of MF-CA3 synapses.

## Discussion

This study uncovers the requirement of TrkB signaling for the establishment of excitatory/inhibitory homeostasis at MFs-CA3 synapses, critical for the development of functional hippocampal circuits. DGCs, via MFs, transfer incoming cortical information in sparse and specific hippocampal signals, which are essential for memory formation. The selective deletion of *Trkb* from immature DGCs early in postnatal development affects the flow of information to the hippocampus proper, thus preventing the establishment of proper synaptic connectivity in targeted neurons. This leads to the disruption of GDPs, a primordial form of synchrony between neurons known to play a critical role in neuronal growth and synaptogenesis (Ben-Ari et al., 2012). Several factors may contribute to altering GDPs in *Trkb*^*Gad1-KO*^ mice, including the early shift of GABA from the depolarizing to the hyperpolarizing direction with a consequent increase in early phasic and tonic GABA_A_-mediated inhibition, resulting in a shunting action at the network level. These changes affect cellular morphology, maturation, and circuit formation downstream of the MFs-CA3 synapses with permanent deficits in hippocampal synaptic plasticity and cognitive functions resembling a neurodevelopmental disorder condition.

Both BDNF and TrkB are expressed at high levels by granule cells and are essential regulators of granule cell morphology (Danzer et al., 2008) (Berg et al., 2019). Here, in particular, we have demonstrated that deletion of *Trkb* from immature DGCs early in postnatal development affects their integration and maturation into the DGC layer possibly due to delayed migration. This phenotype is supported by the accumulation of DCX+ immature granule cells in the SGZ of mutant compared with control mice at 3 weeks age followed by visibly less accumulated DCX+ cells in the SGZ and more tdTomato+ cells in the GCL at 2M age. Moreover, we have studied CREB activation to further support this hypothesis. CREB signaling plays a crucial role in adult hippocampal neurogenesis by regulating the development of newly generated immature DGCs downstream of GABA-mediated excitation (Jagasia et al., 2009). We show that similarly to adult neurogenesis, the peak of CREB activation at P21 in control mice was predominantly detected in the lower third of the DGL at the hilar border where newborn immature neurons reside. Such signal declined by 2M age, coinciding with reduced neurogenesis. In the absence of TrkB signaling, however, there was a reduced activation of CREB at P21 compared with controls followed by a decline at 2M. However, CREB activation at 2M was apparently higher in mutants than in controls, reflecting the delayed migration of DCX+ cells still at this age. Finally, we also show that the morphological maturation of the newly formed DGCs is impaired. Thus, these results support that delayed migration accounts at least in part for the fewer tdTomato cells in the GCL.

BDNF, via TrkB signaling, plays a crucial role in the maturation of inhibition as demonstrated in visual cortical neurons and cultured hippocampal cells (Huang et al., 1999, Yamada et al., 2002). Therefore, it is not surprising that *Trkb* deletion in *Trkb*^*Gad1-KO*^ mice affects GDPs, generated in the immature hippocampus by the synergistic action of glutamate and GABA. The intracellular concentration of Cl^−^, which depends on the developmentally regulated expression of the cation-chloride importer and exporter NKCC1 and KCC2, respectively (Rivera et al., 1999), determines the strength and the direction of GABA_A_-mediated transmission. In immature neurons, increasing BDNF/TRKB signaling *in vitro* promotes the developmental upregulation of KCC2 expression (Aguado et al., 2003), whereas in this study we show that selective *in vivo* reduction of *Trkb* from immature DGCs leads to a hippocampal decrease of *Nkcc1*, in particular, in the CA3 principal cells shown by smFISH. No influence was detected on KCC2 expression. NKCC1 is known to promote network activity in the CA3 hippocampal region (Sipila et al., 2006) and the lack of *Nkcc1* in knockout mice leads, like in our experiments, to GDP disruption (Pfeffer et al., 2009). However, the question is how depletion of BDNF-TrkB signaling from immature DGCs regulates the expression of the chloride importer, *Nkcc1*, in targeted CA3 principal cells. Although how exactly this occurs is still largely unknown and requires further investigation, we provide the following explanations. First, this depletion may affect the integration of excitatory inputs onto DGCs and their activity, as suggested by the reduced number of spines on apical dendrites of these cells. Changes in neuronal activity may disrupt chloride homeostasis known to be particularly sensitive to both activity-dependent processes and BDNF-regulated neuronal activity (Fiumelli and Woodin, 2007). Also, there is evidence that BDNF/TrkB signaling can act as a transsynaptic organizer of neuronal circuits. For example, in mice carrying conditional *Trkb* deletion induced by Synapsin1-Cre strain an increased number of filopodial extensions contacting GABAergic interneurons has been found, which provide feedforward inhibition to CA3 principal cells (Danzer et al., 2008). Consistent with these findings, in the present study, the more selective reduction of *Trkb* in immature DGCs also causes a significant increase in the number of symmetrical (GABAergic) synapses, an effect associated with an enhanced frequency of spontaneous and miniature GABAergic events in the CA3 principal cells. The increased number of GABAergic synapses in our model, *Trkb*^*Gad1-KO*^ mice, may also be related to the selective reduction of the cation-chloride co-transporter NKCC1. In line with our results, previous studies using rat hippocampal cultures (Chudotvorova et al., 2005) and retinotectal neurons of the *Xenopus* visual system (Akerman and Cline, 2006) have demonstrated that a premature reduction of [Cl^−^]_i_ by the early expression of the chloride exporter KCC2 enhances GABAergic innervation.

However, in agreement with the fact that GABAergic input remains shunting onto CA3 hippocampal interneurons throughout development (Banke and McBain, 2006), the increased release of GABA with an equilibrium potential close to the RMP observed in our model would exert a shunting inhibition at the network level with consequent disruption of GDPs leading to severe alterations in the CA3 pyramidal cell dendrites and impairment of synaptic plasticity processes and high cognitive functions. For shunting inhibition, the opening of synaptic and extrasynaptic GABA_A_ receptors by the phasic and tonic release of GABA, respectively, leads to a decrease in membrane resistance with a consequent reduction in the efficacy of excitatory signals that will be unable to reach the threshold for action potential generation (Loscher et al., 2013). Altogether, these data suggest a critical role for TrkB signaling regulating early spontaneous activity, fundamental for the establishment of functional neuronal circuits *in vivo* and animal behavior in adulthood. Moreover, at immature Schaffer collateral-CA1 synapses, GDP-induced LTP strictly depends on BDNF. This acts on presynaptic and postsynaptic TrkB receptors to enhance the probability of glutamate release and to activate the MAPK/ERK signaling pathway, respectively, leading to transcriptional regulation and new protein synthesis in postsynaptic neurons (Mohajerani et al., 2007).

It is renowned that TrkB and its ligand, BDNF, are critical regulators of adult hippocampal synaptic plasticity and learning (Cohen-Cory et al., 2010, Lu et al., 2005, Minichiello, 2009, Musumeci et al., 2009). In particular, in a previous study, deletion of *Trkb* from forebrain principal cells that include the DGCs, starting at around P20 (*Trkb-CaMKII-CRE*) after the majority of granule cells have reached and integrated into the GCL, did not induce morphological and structural defects (Minichiello et al., 1999). Homozygous mutants showed impaired hippocampal LTP and increasingly impaired learning primarily when facing complex or stressful learning paradigms. For example, in contextual FC, *Trkb-CaMKII-CRE* mice showed retarded acquisition of the freezing response by impaired contextual response immediately after the acquisition phase, but proper freezing after 24 h, indicating a short-term synaptic plasticity deficit involving both the hippocampus and proximally connected forebrain structures. In the present study, depletion of TrkB signaling from immature DGCs early in postnatal development impaired morphological maturation of neurons measured by dendritic branching and spine formation with consequent alteration of adult hippocampal synaptic plasticity and learning. In the FC paradigm mutants showed decreased acquisition of the freezing response during the training phase and impaired recognition of the neutral context after 24 h, but a normal response to a neutral stimulus (tone), suggesting hippocampal-dependent long-term memory deficit. Thus, in contrast to adult *Trkb* deletion, removing TrkB signaling in immature DGCs early in postnatal development affects neurodevelopmental programs leading to adult neural dysfunction. Although the Cre-mediated deletion of *Trkb* in the present model is selective within the hippocampus, it does occur in some GABAergic neurons of other brain areas (Ohtsuka et al., 2013). Therefore, we cannot exclude the possibility that alterations in synaptic strength can involve other brain areas connected to the hippocampus proper such as the EC and the amygdala. However, the requirement for TrkB signaling in immature DGCs is cell autonomous as indicated by genetically based experiments wherein postnatal deletion of *Trkb* from forebrain principal neurons including differentiated DGCs showed impaired spatial learning and synaptic plasticity, but normal brain morphology (Minichiello et al., 1999). Also, specific ablation of TrkB signaling from adult dentate neural progenitor cells (NPCs) using a Cre line enabling deletion in radial glia progenitor cells affects survival and maturation of NPCs and results in impaired response to antidepressant treatment. The latter does not occur if *Trkb* is deleted from the differentiated DGCs (Li et al., 2008, Minichiello et al., 1999).

Overall, this study suggests that early in postnatal development BDNF-TrkB activation in immature DGCs provides an instructive signal that drives the sequential maturation of intrinsic hippocampal circuits via the depolarizing action of GABA. Disrupting this signal at a critical period leads to neurodevelopmental defects, followed by synaptic plasticity and cognitive impairments in adulthood. The changes we have observed in *Trkb*^*Gad1-KO*^ mouse model are recurrent pathological features shared in part by many neurodevelopmental disorders (Griguoli and Cherubini, 2017) (Deidda et al., 2014). Moreover, the present data establish the genetic importance of TrkB signaling at a critical period, shaping hippocampal neuronal circuitry by controlling the GABAergic system development. Therefore, these findings have clinical implication by revealing the existence of sensitive time window in which a developing brain might be more amenable to treatment and may stimulate further research into mechanisms underlying this type of disorders.

### Limitations of the Study

Our data establish the genetic importance of TrkB signaling in immature DGCs driving the sequential development of intrinsic hippocampal circuits by modulating early GABA signaling through the expression of *Nkcc1*. It would be interesting to determine if by rescuing *Nkcc1* expression in the postsynaptic target of DGCs, the CA3 principal cells, would be sufficient to revert or prevent the phenotype. Moreover, it would also be instrumental to determine the exact mechanism whereby presynaptic TrkB signaling (DGCs) affects postsynaptic *Nkcc1* expression levels (CA3 principal cells).

## Methods

All methods can be found in the accompanying Transparent Methods supplemental file.

## Data and Code Availability

This study did not generate datasets or code.

## Supplemental Information

Document S1. Transparent Methods, Figures S1–S7, and Table S4

Table S1. Statistical Analysis for Data Presented in Figures S6A and S6B and Related to Figure 6 (A and B) Paired-pulse facilitation of fEPSPs evoked at the CA3-CA1 synapse following stimulation of Schaffer collaterals.

Table S2. Statistical analysis for Data Presented in Figures 6B and 6C (B) Time course of changes in fEPSP slopes (mean ± SEM) following high-frequency stimulation of Schaffer collaterals. (C) fEPSP data (mean ± SEM) included in each histogram.

Table S3. Statistical Analysis for Data Presented in Figure S6D and S6E and Related to Figure 6 (D) Time course of changes in fEPSP slopes (mean ± SEM) following high-frequency stimulation of Schaffer collaterals. (E) fEPSP data (mean ± SEM) included in each histogram.
