# Supplementary material for: Immature Dentate Granule Cells Require Ntrk2/Trkb for the Formation of Functional Hippocampal Circuitry
Source: iScience. 2020 Apr 18;23(5):101078. doi: 10.1016/j.isci.2020.101078 (PMC7200316; doi:10.1016/j.isci.2020.101078)
Supplement: Document S1. Transparent Methods, Figures S1–S7, and Table S4 [file mmc1.pdf]

iScience, Volume *23*

Supplemental Information

Immature Dentate Granule Cells Require *Ntrk2*/*Trkb* for the Formation of Functional Hippocampal Circuitry

Sylvia Badurek, Marilena Griguoli, Aman Asif-Malik, Barbara Zonta, Fei Guo, Silvia Middei, Laura Lagostena, Maria Teresa Jurado-Parras, Thomas H. Gillingwater, Agnès Gruart, José María Delgado-García, Enrico Cherubini, and Liliana Minichiello

Supplemental Figures

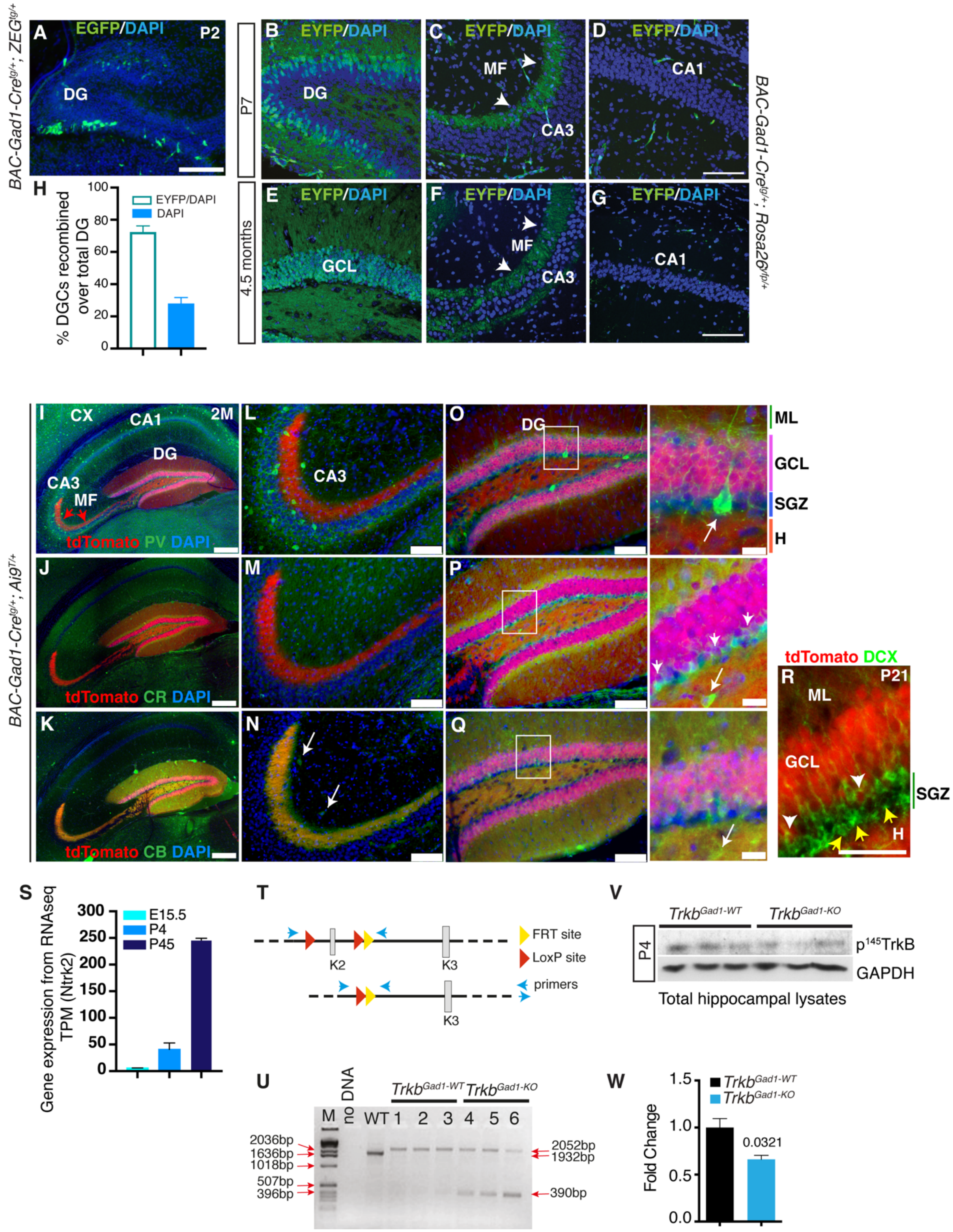

Figure S1. ***Gad1-Cre*** **recombination is specific to immature dentate granule cells within the hippocampus. Related to Figure 1.**

(**A-R**) BAC-*Gad1-Cre* line expression pattern within the hippocampal formation revealed by crossing the Cre line to various reporter strains. (**A**) Representative sagittal hippocampal

sections from a P2 *BAC-Gad1-Cre*$^{tg/+}$*; Z/EG*$^{tg/+}$ mouse. The reporter signal, EGFP (green), and DAPI-stained nuclei (blue) were superimposed. (**B-H**) Representative sagittal hippocampal sections from P7 (B-D) and 4.5M (E-G) *BAC-Gad1-Cre*$^{tg+/}$*; R26R*$^{yfp/+}$ mice. The reporter signal, EYFP (green), and DAPI-stained nuclei (blue) were superimposed. In the CA3 region, mossy fibres (arrowheads) show the EYFP signal as a result of the recombination in the dentate gyrus granule cells.

(**H**) Quantification of EYFP-expressing cells versus total DG cells in adulthood (4.5M). Total DG cells were identified by DAPI staining. The percent of EYFP positive DGCs over the total number of DAPI-stained DG nuclei: 72 ± 4% SEM. N=3 mice (on average 2 sections per mouse were used).

(**I-K**) Representative coronal sections from 2M old *BAC-Gad1-Cre*$^{tg/+}$*; Ai9R/+* mouse brain (n=2), highlighting prominent tdTomato fluorescent signal (red) in dentate granule neurons and the MF terminals (arrowheads), were immunostained with primary antibodies against Parvalbumin (PV), Calretinin (CR), and Calbindin (CB), (green) respectively. The green signal was superimposed to tdTomato and DAPI signals. No colocalization was observed between each primary antibody (green) and tdTomato signal in interneurons (arrows) both in the CA3 region and the DG (**L-Q** and respective insets); however, a colocalization was observed between tdTomato signal and Calretinin (green) in immature granule cells within the SGZ (**P** and inset, arrowheads), and as expected, between tdTomato and Calbindin (green) in mature granule cells in the GCL (**Q** and inset).

(**R**) Representative coronal hippocampal sections from a P21 *BAC-Gad1-Cre*$^{tg+/}$*;* Ai9$^{T/+}$ mice. tdTomato reporter signal (red) and DCX immunostaining (green) were superimposed. White arrowheads indicate examples of colocalization between tdTomato and DCX occurring only in immature neurons (DGCs beginning to migrate and mature in the GCL) but not in neural precursors (yellow arrows).

(**S**) Gene expression data expressed in transcripts per million (TPM) and extracted from '*Table 2' in* BERG ET AL., 2019, (Berg et al., 2019) showing developmental upregulation of *Ntrk2/Trkb* in dentate neural progenitors at different stages (E15.5, P4 and P45); qval 0.0456 for E15.5 vs P4; and 0.0051 for P4 vs P45.

(**T-U**) PCR strategy to detect *Trkb* recombination. (**T**) Schematic representation of *Trkb* floxed allele before and after Cre-mediated recombination. (**U**) *Trkb* recombination by PCR strategy. Genomic DNA was isolated from adult hippocampus. The strategy chosen amplifies a PCR product of 2052bp from the non-recombined *Trkb* floxed allele, a 1932bp from the wild-type *Trkb* allele, and a 390bp from the *Trkb* floxed allele after Cre-mediated recombination. The recombined band (390bp) was detected in every sample from *Trkb*$^{Gad1\text{-}KO}$ mice together with a faint 2052bp band for the un-recombined allele. Control mice, which carry *Trkb* floxed alleles but not *Gad1-Cre* transgene, showed only the larger PCR products resulting from the unrecombined *Trkb* floxed allele. The WT sample showed only a wild-type *Trkb* allele size band (1932bp).

(**V-W**) Western blot analysis (V) and quantification of fold changes (W) from P4 total hippocampal lysates shows significantly reduced TrkB full length in *Trkb*$^{Gad1\text{-}KO}$ mice compared to *Trkb*$^{Gad1\text{-}WT}$ (*Trkb*$^{Gad1\text{-}WT}$, n=3: 1.000 ± 0.09576, *Trkb*$^{Gad1\text{-}KO}$, n=3: 0.6615 ± 0.04303, *P*=0.0321).

DG, dentate gyrus; MF, mossy fibres, GCL, granule cell layer; CX, cortex; DG, dentate gyrus, MF, mossy fibers. ML, molecular layer; GCL, granular cell layer; SGZ, subgranular zone; H, hilus. DCX, Doublecortin. Scale bars A-G (100µm); I-K (250µm); L-Q (100µm); insets (10µm); R (25µm).

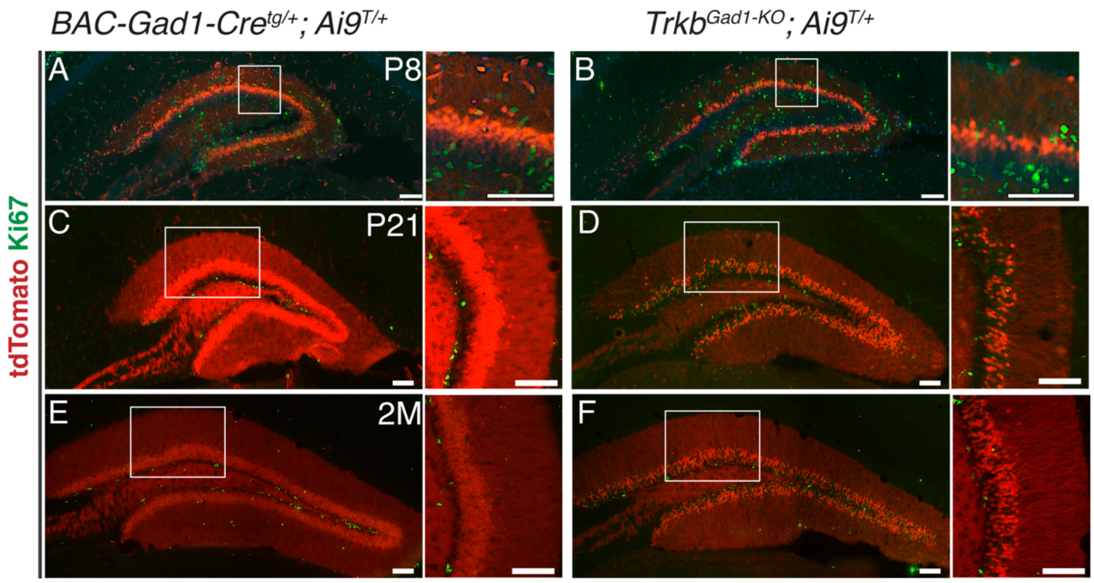

Figure S2. Similar proliferation of dentate progenitors between mutants and controls. Related to Figure 2.

(**A-F**) Immunostaining for Ki67 (green) shows similar pattern of dentate progenitor proliferation between controls (*BAC-Gad1-Cre*$^{tg/+}$*; Ai9*$^{T/+}$) and mutants (*Trkb*$^{Gad1\text{-}KO}$*; Ai9*$^{T/+}$) at all ages analysed, P8 (**A-B**, and respective insets), P21 (**C-D**, and respective insets) and 2M (**E-F**, and respective insets). Scale bars, A-F, and respective insets (100μm).

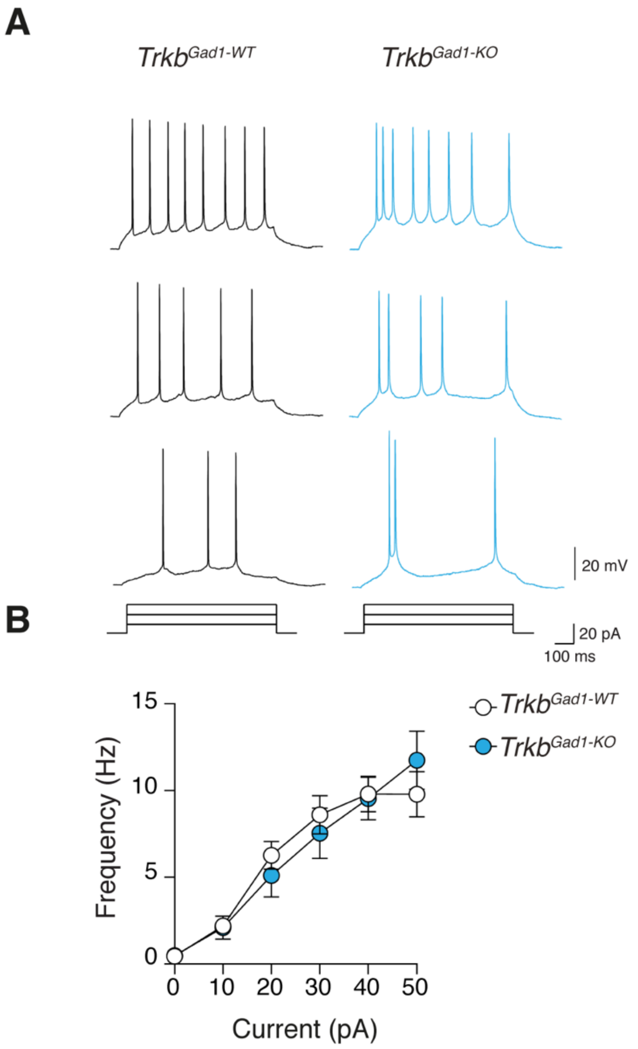

Figure S3. Neuronal excitability is unchanged in *Trkb*$^{Gad1\text{-}KO}$ mice. Related to Figure 3.
(**A**) Sample traces showing the firing activity induced in CA3 principal cells by depolarizing current pulses of increasing amplitude (delivered in the presence of AMPA, NMDA and $GABA_A$ receptor antagonists) from control littermate and *Trkb*$^{Gad1\text{-}KO}$ mice.
(**B**) Each point represents the mean spike frequency (Hz) versus the amount of injected current (pA) in control and in *Trkb*$^{Gad1\text{-}KO}$ mice. No significant differences were observed between the two genotypes.

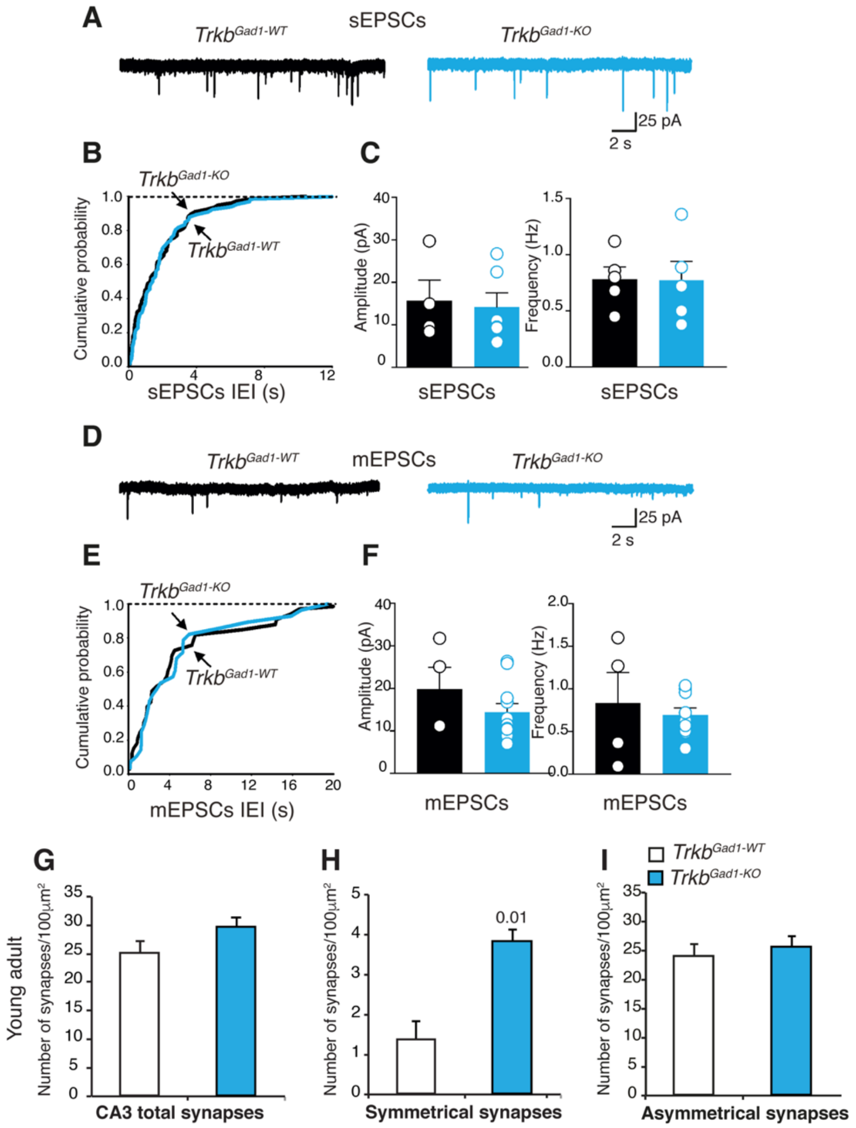

Figure S4. Spontaneous and miniature AMPA-mediated postsynaptic currents (sEPSCs and mEPSCs) are not affected in *$Trkb^{Gad1\text{-}KO}$* mice. Related to Figure 3.

(**A**) sEPSCs recorded in isolation (in the presence of bicuculline, DL-APV to block $GABA_A$ and NMDA receptors, respectively) from CA3 principal cells in hippocampal slices from P5-P9 in control ($Trkb^{Gad1\text{-}WT}$, black) and in $Trkb^{Gad1\text{-}KO}$ mice (cyan).

(**B**). Cumulative distribution of inter event interval (IEI) for cells shown in (A).

(**C**). Summary plots of mean amplitude and frequency of sEPSCs shown in (B); sEPSCs [amplitude, $Trkb^{Gad1\text{-}WT}$, 17 ± 4 pA (n=5 from 2 pups) vs. $Trkb^{Gad1\text{-}KO}$, 14 ± 3 pA (n=7 from 5pups), P>0.05]; [frequency, $Trkb^{Gad1\text{-}WT}$, 0.78 ± 0.1 Hz (n=5 from 2 pups) vs. $Trkb^{Gad1\text{-}KO}$, 0.77 ± 0.17 (n=7 from 5 pups), P>0.05].

(**D-F**) as in (A-B) but for mEPSCs [amplitude, $Trkb^{Gad1\text{-}WT}$, 20 ± 8 pA (n=4 from 2 pups) vs. $Trkb^{Gad1\text{-}KO}$, 14 ± 3 pA (n=11 from 5 pups), P>0.05]; [frequency, $Trkb^{Gad1\text{-}WT}$ 0.83 ± 0.35 Hz (n=4 from 2 pups) vs. $Trkb^{Gad1\text{-}KO}$ 0.69 ± 0.25 Hz (n=11 from 5 pups), P>0.05]. Values are mean ± SEM. P statistic from unpaired Student's t-tests.

(**G-I**) Symmetrical synapses remain increased in the young adult Trkb$^{Gad1\text{-}KO}$ CA3 hippocampal region. Number of synapses in the CA3 hippocampal region/100μm^2, two months old mice (young adult). (**G**) Total (asymmetric and symmetric), *Trkb*$^{Gad1\text{-}WT}$, 24.93 ± 2.18, *Trkb*$^{Gad1\text{-}KO}$, 29.56 ± 1.88, P>0.05. (**H**) Symmetrical synapses, *Trkb*$^{Gad1\text{-}WT}$, 1.34 ± 0.47 vs. *Trkb*$^{Gad1\text{-}KO}$, 3.83 ± 0.29, P=0.01. (**I**) Asymmetrical synapses, *Trkb*$^{Gad1\text{-}WT}$, 24.00 ± 2.46 vs *Trkb*$^{Gad1\text{-}KO}$, 25.74 ± 1.95, P>0.05. *Trkb*$^{Gad1\text{-}WT}$ (n=6), *Trkb*$^{Gad1\text{-}KO}$ (n=3). Values are mean ± SEM, P statistic from unpaired one-tailed Student's t-tests.

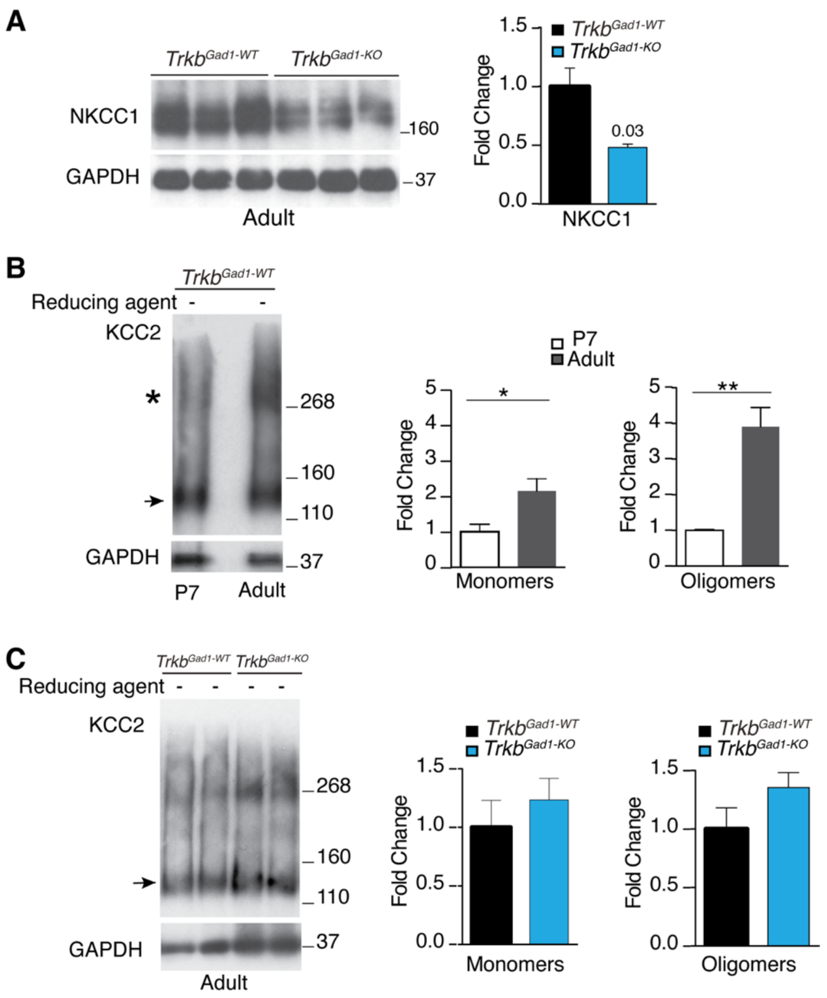

Figure S5. NKCC1 but not KCC2 expression is altered in the hippocampus of adult *Trkb$^{Gad1-KO}$* mice. Related to Figure 4.

(**A**) Representative western blots (WB) from adult (P50) hippocampal lysates and relative quantification of NKCC1 protein levels [P50 Control (*Trkb$^{Gad1-WT}$*) (n=6), 1.000 ± 0.1557, *Trkb$^{Gad1-KO}$* (n=6): 0.4815 ± 0.02869, *P*=0.03].

(**B-C**) Representative WB from P7 and P50 hippocampal protein lysates and relative quantification of KCC2 monomers and oligomers. (**B**) Protein lysates were not treated with a reducing agent and were separated by a linear 3-8% Tris-acetate NuPAGE gel to detect KCC2 oligomers. Distinct KCC2 immunoreactive bands around 130 and 270 kDa were detected mainly at P50. Arrow denotes the monomeric KCC2, and the asterisk points to KCC2 oligomers. Quantification reveals significant developmental increase between P7 and P50 of both monomers (*Trkb$^{Gad1-WT}$*, P7 (n=3), 1.000 ± 0.2142; *Trkb$^{Gad1-WT}$*, P50 (n=3), 2.157 ± 0.3415, *P*=0.045) and oligomers (*Trkb$^{Gad1-WT}$*, P7 (n=3), 1.000 ± 0.02456; *Trkb$^{Gad1-WT}$* P50 (n=3), 3.909 ± 0.5392, *P*=0.0057). (**C**) Quantification of KCC2 monomers and oligomers at P50 between mutants and controls (monomers, *Trkb$^{Gad1-WT}$* (n=3), 1.000 ± 0.2306; *Trkb$^{Gad1-KO}$* (n=3), 1.234 ± 0.1837, *P*=0.47; oligomers, *Trkb$^{Gad1-WT}$* (n=3), 1.000 ± 0.1810; *Trkb$^{Gad1-KO}$* (n=3), 1.355 ± 0.1273, *P*=0.18). GAPDH was used as loading control. Values are mean ± SEM. *P* statistic from unpaired Student's *t*-test.

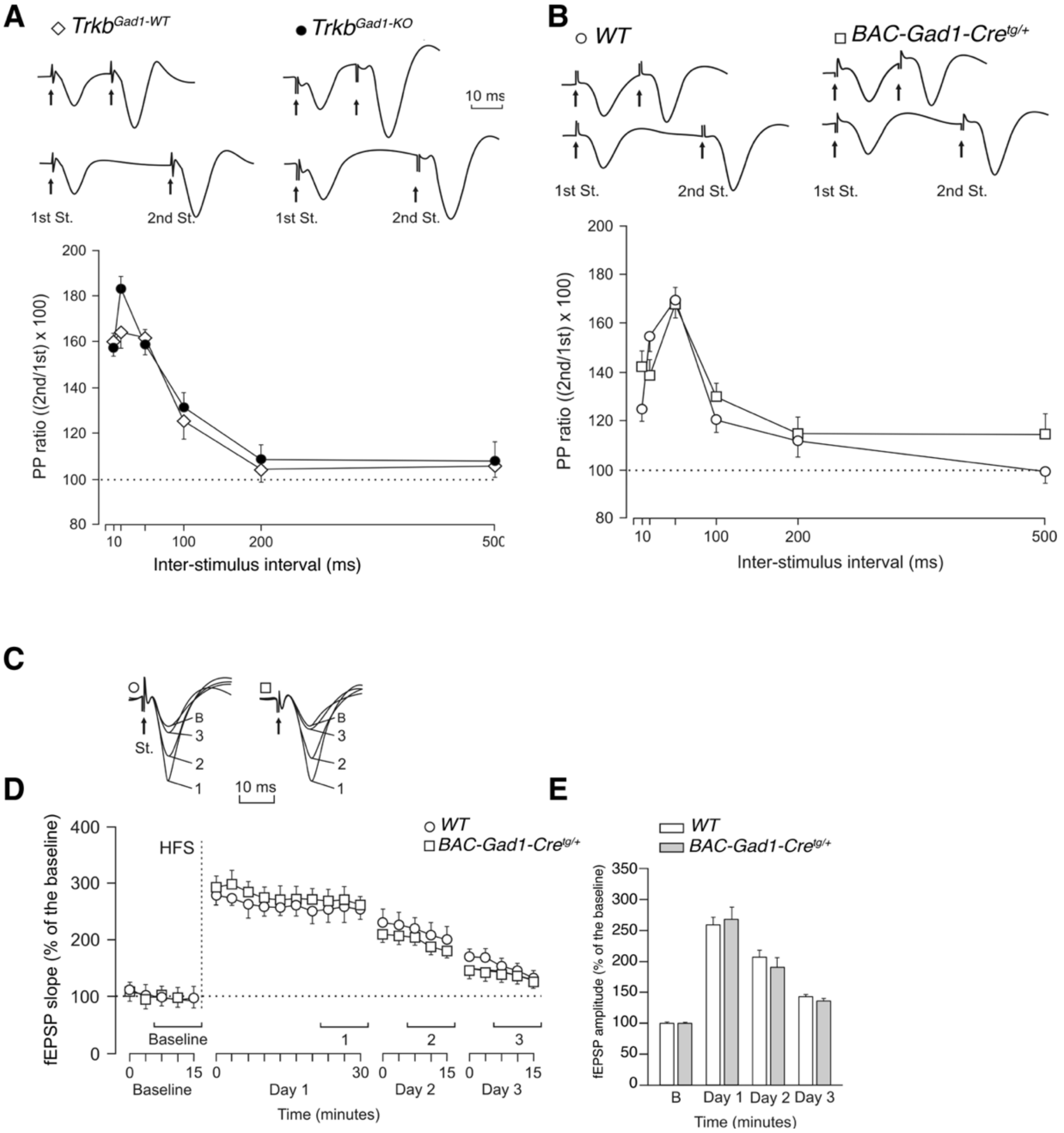

Figure S6. *In vivo* presynaptic short-term plastic properties are normal at CA3-CA1 synapses in *Trkb*$^{Gad1\text{-}KO}$ mice. Related to Figure 6; Table S1. Statistical analysis for data presented in this Figure S6A-B and related to Figure 6; Table S2. Statistical analysis for data presented in Figure 6B-C. Table S3. Statistical analysis for data presented in this Figure S6D-E and related to Figure 6. (**A**) Paired-pulse facilitation of fEPSPs evoked at the CA3-CA1 synapse following stimulation of Schaffer collaterals. Representative examples of fEPSP paired traces collected for the *Trkb*$^{Gad1\text{-}WT}$ and *Trkb*$^{Gad1\text{-}KO}$ groups at 20ms and 40ms inter-stimulus intervals. Represented values are mean ± SEM slopes of the second fEPSP expressed as a percentage (%) of the first for the six inter-stimulus intervals. The two groups presented a significant ($P \leq 0.038$) increase in the response to the 2nd pulse at short (10-40ms) time intervals, with the largest differences being reached at 40ms of inter-pulse intervals. No differences were observed in their facilitation curves [$F_{(5,70)} = 0.146$, $P=0.980$; n=8 animals per group. Two Way Repeated Measures ANOVA, One Factor Repetition]. (**B**) Similar analysis was carried out for the additional control groups (*BAC-Gad1-Cre*$^{tg/+}$ and WT littermates). Also

in this case, the two groups presented a significant ($P \leq 0.023$) increase of the response to the 2nd pulse at short (20-40ms) time intervals, with the largest differences being reached at 40ms of inter-pulse intervals, but no differences in their facilitation curves [$F_{(5,70)} = 0.916$, P=0.476; n=8 animals per group. Two Way Repeated Measures ANOVA, One Factor Repetition]. (**C**) Representative examples fEPSP recordings collected from selected animals of each experimental group at times indicated in (D). In all cases, the smaller fEPSP was collected before the HFS (baseline), while the larger one was collected 24-30min after the HFS. (**D**) Time course of changes in fEPSP slopes (mean ± SEM) following HFS stimulation of Schaffer collaterals. The HFS was presented after 15 min of baseline recordings at the time marked by the dashed line. fEPSPs are given as a percentage of baseline (100%) values. Following the HFS session, fEPSPs were recorded for 30min on day 1, and for 15min on the following days. No significant differences were observed between groups [$F_{(24,336)} = 1.343$; P=0.133; n=8 animals per group. Two Way Repeated Measures ANOVA, One Factor Repetition]. (**E**) fEPSP data (mean ± SEM) included in each histogram (B, Baseline; Day 1, 2, 3) were collected for the time intervals indicated in (D). No significant differences were observed between these two groups [$F_{(3,138)} = 2.166$; $P = 0.095$; n=8 animals per group. Two Way Repeated Measures ANOVA, One Factor Repetition].

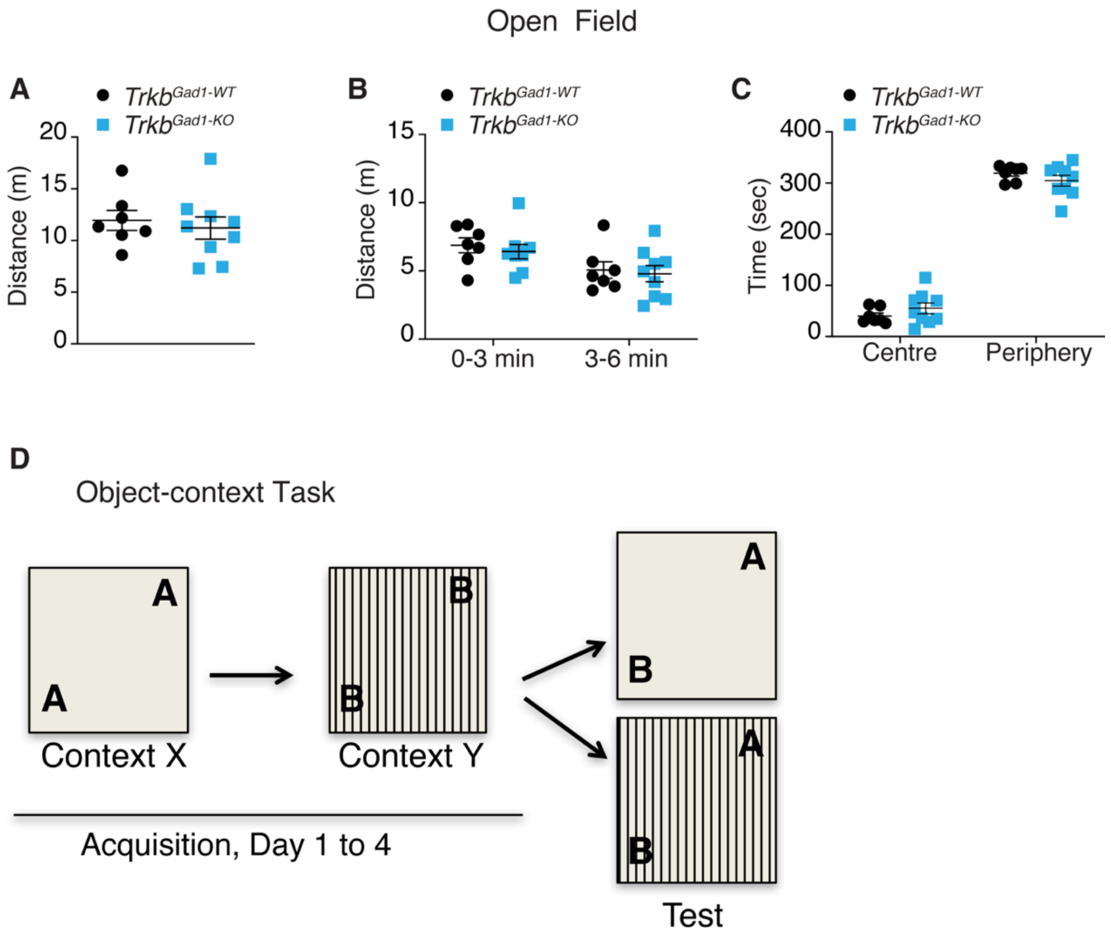

Figure S7. Open field (OF) and object-context recognition (OCR) task. Related to Figure 7. (**A**) Six-month-old mice were tested in the OF; distance travelled, *$Trkb^{Gad1-WT}$* (n=7) and *$Trkb^{Gad1-KO}$* (n=8) mice, in a 6-min session of the OF. No difference was found between genotypes (mean distance ± SEM*;* *$Trkb^{Gad1-WT}$*, 11.22±1.08; *$Trkb^{Gad1-KO}$*, 11.96±0.97, unpaired Student's *t*-test: $t_{(14)}$= 0.4947, *P*=0.6285). (**B**) In the OF, both *Trkb* mutant and control mice travelled a shorter distance in the second half compared to the first half of the 6-min session, suggesting habituation to the arena (Two-way ANOVA, repeated measures, $F_{(1,14)}$= 28.88, *P*<0.0092, main effect of time). (**C**) *$Trkb^{Gad1-KO}$*, similar to *$Trkb^{Gad1-WT}$* mice, spent less time in the centre compared to the periphery of the open field, suggesting that they were not more anxious than controls (two-way ANOVA; main effect of position: $F_{(1,28)}$= 842.9, *P*<0.0001). (**D**) OCR test experimental design. The object-context test was performed according to previous studies (Wilson et al., 2013). Acquisition phase, day 1 to day 4, mice were exposed to two copies of object A in context X and two copies of object B in context Y, 5 min a day for four days. The order of the exposure to the two contexts was alternated on each day (e.g., X-Y, Y-X, X-Y, Y-X). Day 5, test, mice received a 3 min test trial in one of the two contexts (depending on the order of their exposure to the contexts during the acquisition phase). A preference for the object that was not previously paired with the context indicated context-dependent object recognition memory. During the test phase, objects consisted of exact copies of those explored during the acquisition phase. The identity of the objects, their location, and the order of test trials in context X and Y was fully counterbalanced within each genotype.

Table S4. Probe sets for *Nkcc1* smFISH analysis. Related to Figure 4E-G.

		**Nkcc1 (mouse)**		Probe set name: Nkcc1_555
		**Fluorophore: Quasar 570, 548 EX (NM), 566 EM (NM)**		
Count 48	PROBE #	PROBE (5' > 3')	PROBE POSITION *	PERCENT GC
	1	tcatagtagtactgctggtg	568	45.00%
	2	aagccatcctcaaaaggttc	739	45.00%
	3	atctctggttggagtacttt	773	40.00%
	4	tacaactcctttactctcgg	812	45.00%
	5	cggactaatacacccttgat	844	45.00%
	6	aaagagcatcacaccccaaa	878	45.00%
	7	ccacgatccatgacaatcta	900	45.00%
	8	ccattgctattacgacgaca	942	45.00%
	9	gctgaagtagacaatcctgt	982	45.00%
	10	caacatacatagcaacggcc	1116	50.00%
	11	tggaatgttccttaagcagc	1161	45.00%
	12	cgactgtaatggctccaata	1215	45.00%
	13	ctatgacgaagtcggcgatg	1317	55.00%
	14	tccagctagaataccagttg	1475	45.00%
	15	tatggctgactgaggatctg	1517	50.00%
	16	catcccgaacaacacacgaa	1605	50.00%
	17	tgtcagctctgttgtaatgg	1646	45.00%
	18	gttcattagcccgtaagaac	1727	45.00%
	19	gacgagagcgtggctgaaaa	1804	55.00%
	20	gggagcactcacaagagatg	1832	55.00%
	21	ctgggtagatattgtcctta	1872	40.00%
	22	tttcccataacctttagcaa	1904	35.00%
	23	aaccacgaagcggttcatta	1926	45.00%
	24	gaatcccagtgcaatcagaa	1958	45.00%
	25	ctccaataagggagatccac	2121	50.00%
	26	aaatgtacagcccaaggact	2205	45.00%
	27	agtgcactgaggtaagtcag	2275	50.00%
	28	ccccagaaagacggattgaa	2301	50.00%
	29	gagtttggagaacctgtcat	2362	45.00%
	30	ggcttgatcaatggacatct	2483	45.00%
	31	catcaagtattgtgcacctt	2576	40.00%
	32	acaagtgtgtttggcttcat	2617	40.00%
	33	atatacatatccacatccct	2674	35.00%
	34	tccacatttactaccacatc	2827	40.00%
	35	aagcttttggtcagctacat	2987	40.00%
	36	gaaactgtgtgctagcttca	3009	45.00%
	37	aagccaccagacatctatag	3050	45.00%
	38	atgtcaaacctccatcatca	3072	40.00%

39	ataaagtagccatcgctctc	3189	45.00%
40	atcagagaagtctatccgga	3218	45.00%
41	ttgatatctccaaggaccat	3241	40.00%
42	ggctcgatcatgtcatcaaa	3292	45.00%
43	ttatctgtgattcgccaagg	3373	45.00%
44	acctgatctgtcgatatgtc	3417	45.00%
45	gcgactggaagactcatgac	3481	55.00%
46	ccatatacagagcactggac	3516	50.00%
47	ttcgagagagcttctaacca	3538	45.00%
48	gtagaaggtaaggacgctct	3596	50.00%

KEY RESOURCES TABLE

REAGENT or RESOURCE	SOURCE	IDENTIFIER
**Antibodies _Biochemistry**		
TrkB	Cell Signaling	#4603
KCC2	Upstate	07-432
NKCC1	Sigma-Aldrich	T4-S
GAPDH	Sigma-Aldrich	#G954S
HRP Goat anti-rabbit	Stratech Jackson	111-035-008
HRP Donkey anti-goat HRP	Stratech Jackson	705-035-003
HRP Goat anti-mouse rabbit	Stratech Jackson	115-035-003
**Antibodies for immunohistochemistry**		
Calbindin	Sigma	CB995
Calretinin	Swant	7699/4
Parvalbumin	Swant	PV-25
Phospho-Creb (Ser133)	Cell Signaling	87G3
Ki67	Abcam	ab 15580
DCX	Abcam	ab113435
Chicken Anti-GFP	Abcam	ab 13970
Alexa Fluor® 555 Donkey anti-rabbit	Invitrogen	A-31572
Alexa Fluor® 555 Donkey anti-mouse	Invitrogen	A-31570
Alexa Fluor® 488 AffiniPure Donkey Anti-Chicken	Jackson Immunoresearch	703-545-155
Biotinylated goat anti-rabbit	Vector Labs	BA-1000
Biotinylated horse anti-rmouse	Vector Labs	BA-2000
**Experimental Models: Organisms/Strains**		
Trkb-floxed (*Trkb*^{lx}) strain	Minichiello et al., 1999	NA
BAC-Gad1-Cre$^{tg/+}$ strain	Ohtsuka et al., 2013	NA
Trkb$^{Gad1\text{-}KO}$ mice	This paper	NA
Z/EG reporter strain	Novak et al., 2000	NA
Rosa26-EYFP	Srinivas et al., 2001	NA
Rosa26-Ai9-tdTomato	Jackson Laboratory	Jax Stock#007909
**Oligonucleotides**		
smFISH probes for *Nkcc1*	This paper	Attached as a table

TRANSPARENT METHODS

Experimental model and subject details

All animal procedures conformed to the UK legislation Animals (Scientific Procedures) Act 1986 and to the University of Edinburgh Ethical Review Committee (ERC) policy as well as the University of Oxford ERC policy, and to the National and International laws and policies (EEC Council Directive 86/609, OJ L 358, 1, December 12, 1987; NIH Guide for the Care and Use of Laboratory Animals, NIH Publication No. 85-23, 1985 revised in 1995) procedures at the European Molecular Biology Laboratory (Mouse Biology Unit) Italy, the International School for Advanced Studies (SISSA), Trieste, and European Brain Research Institute (EBRI), Rome, Italy. To European Union Council (2003/65/EU) and Spanish (BOE 252/34367-91, 2005) guidelines for the use of laboratory animals in chronic electrophysiological and behavioural studies as well as the Ethics Committee of the Pablo de Olavide University, Seville, Spain. For all experiments, we used male and female mice, unless stated otherwise, at the age and numbers specified throughout the text.

Mouse strains

$Trkb^{Gad1\text{-}KO}$ mice, this line derives from a cross between the *Trkb*-floxed ($Trkb^{lx}$) line, previously described (Minichiello et al., 1999), and the $BAC\text{-}Gad1\text{-}Cre^{tg/+}$ strain, previously described (Ohtsuka et al., 2013). Littermates without the Cre transgene were used as controls ($Trkb^{lx/lx}$; $Gad1\text{-}Cre^{+/+}$) unless specified. However, to ensure that the transgene did not carry any phenotype, mice carrying only the transgene ($BAC\text{-}Gad1\text{-}Cre^{tg/+}$) and wild-type control littermates were also analysed (see Fig.S6, Table S1, Table S3). Moreover, no differences were found between wild-type and *Trkb* floxed animals. The reporter lines used were the Z/EG (Novak et al., 2000), the Rosa26-EYFP (Srinivas et al., 2001), and the Rosa26-Ai9-tdTomato (Madisen et al., 2010). All experiments were carried out by an experimenter blind to genotype.

Group allocation

Littermates were grouped according to their genotypes and then used for all different experiments. Masking was used during group allocation, data collection and data analysis for all electrophysiological and behavioural tests.

***In vitro* electrophysiology**

Experiments were performed on hippocampal slices from neonatal (P3-P9) and adult (P60-P90) mutants and control littermate mice. For neonatal mice, each experiment was replicated minimum 3 times and 3-4 weeks apart (with at least 3 biological replicates and 3 technical replicates). For the adult stage, slices were prepared from 3 to 5 mice for each experimental group. More details are included in the figure legends.

Slice preparation

Briefly, animals were decapitated and the brain quickly removed from the skull and placed in ice-cold artificial cerebrospinal fluid (aCSF) solution containing (in mM): NaCl 130, KCl 3.5, NaH_2PO_4 1.2, $NaHCO_3$ 25, $MgCl_2$ 1.3, $CaCl_2$ 2, glucose 25, saturated with 95% O_2 and 5%

CO_2 (pH 7.3-7.4). Transverse hippocampal slices (300µm thick) were cut with a vibratome and stored at room temperature in a holding bath containing the same solution as above. After a recovery period of at least one-hour, an individual slice was transferred to the recording chamber where it was continuously superfused with oxygenated aCSF at a rate of 2-3ml/min at 33-34°C.

Electrophysiological recordings

In neonatal animals, electrophysiological experiments were performed from visually identified CA3 pyramidal cells using the whole-cell configuration of the patch-clamp technique in current or voltage-clamp mode. Patch electrodes were pulled from borosilicate glass capillaries (Hingelberg, Malsfeld, D). They had a resistance of 4-6 MΩ when filled with an intracellular solution containing (in mM) KCl 125, NaCl 10, HEPES 10, MgATP 4, Na-GTP 0.4, EGTA 0.5. Recordings were made with a patch clamp amplifier (Multiclamp 700B; Molecular Devices, Sunnyvale, CA, USA). Series resistance compensation was used only for current-clamp recordings. The stability of the patch was checked by repetitively monitoring the input and series resistance during the experiment. Cells exhibiting more than 20% changes in series resistance were excluded from the analysis. Spontaneously occurring GDPs were recorded in current clamp conditions from a holding potential of -70 mV. Spontaneous and miniature AMPA (sEPSCs) and $GABA_A$-mediated postsynaptic currents (GPSCs) were recorded in voltage clamp conditions from a holding potential of -70 mV, using the intracellular solution mentioned above. Miniature EPSCs and GPSCs were recorded in the presence of tetrodotoxin (TTX, 1µM, purchased from Latoxan, Valence, France). Spontaneous and miniature events were analysed with pClamp 9 (Molecular Devices, Sunnyvale, CA, USA). This program uses a detection algorithm based on a sliding template. The template did not induce any bias in the sampling of events because it was moved along the data trace by one point at a time and was optimally scaled to fit the data at each position. All the collected events were averaged, and the peak of the mean current amplitude was calculated.

The effect of isoguvacine on cells firing was studied in cell-attached recordings. In these cases, the patch pipette was filled with aCSF. Isoguvacine was applied from a puff pipette (intrapipette concentration of isoguvacine, 100µM dissolved in aCSF) positioned close to the patched neurons using a pneumatic PicoPump [PV820 (World Precision Instruments); pulse duration, 0.5–1 s; pressure, 6-8 psi]. To measure the effect of isoguvacine on neuronal firing, we calculated the ratio between the mean firing frequency during 30s windows preceding and following the application of the drug.

In adult animals, field (f)EPSPs were obtained using a glass microelectrode filled with aCSF placed in the *stratum lucidum* of the CA3 area. fEPSPs were evoked at the frequency of 0.1 Hz with bipolar twisted NiCr-insulated electrodes (50µm o.d.), positioned on the mossy fibre tract close to the recording electrode. The duration of the stimulus was 80-100µs; the stimulation intensity corresponded to that necessary to obtain a response equal to 50% of the maximal fEPSP. Paired responses evoked 50ms apart were used to measure the paired-pulse ratio (PPR). PPR was calculated as the mean amplitude of the synaptic response evoked by the second stimulus over that evoked by the first one. The input-output curves were constructed by plotting

the amplitude of the response *vs* stimulation intensity. LTP was induced by theta burst stimulation of mossy fibres (10 trains, 200Hz, 50ms each at a rate of 1/100ms). Recordings were obtained with a Multiclamp 700B amplifier connected to a Digidata 1550. Data were acquired with pClamp 9.2 (Molecular Devices), digitised at 10kHz, filtered at 3kHz, and analysed offline with Clampfit 9.2 (Molecular Devices).

Gramicidin-perforated patch recordings

GABA-mediated postsynaptic currents (GPSCs) evoked in CA3 principal cells by stimulation of MFs in *stratum lucidum* (in the presence of DNQX and DL-APV to block NMDA and AMPA receptors, respectively) were recorded (from a holding potential of -70 mV) using gramicidin perforated patch. In this case, the patch pipette solution contained (in mM): 150 KCl and 10 HEPES, buffered to pH7.2 with Tris-OH. The extracellular solution contained (in mM): NaCl 130, KCl 3.5, NaH2PO4 1.2, NaHCO3 25, MgCl2 1.3, CaCl2 2, glucose 25, saturated with 95% O2 and 5% CO2 (pH 7.3-7.4). Gramicidin was first dissolved in DMSO to prepare a stock solution of 10–40mg/ml and then diluted in the pipette solution to a final concentration of 80 μg/ml. The gramicidin-containing solution was prepared and sonicated <1 h before the experiment. To facilitate cell-attached formation (4–10GΩ), patch pipettes were backfilled with a gramicidin-containing solution. Recordings started 20-30 min after formation of the cell-attached seal and stabilization of the series resistance (*R*s) around 60MΩ. Depolarizing currents were intermittently injected to evoke action potentials to verify the patch integrity, and in the case of membrane rupture, the recording was discontinued. At the end of each recording, negative pressure was applied to break the membrane and establish whole-cell configuration. This was associated with a shift of the reversal potential of the GABA-mediated responses to near 0mV.

Drugs used: 6,7-dinitroquinoxaline-2,3-dione (DNQX), bicuculline (Ascent Scientific), isoguvacine (Sigma-Aldrich), glycine, N-methyl-D-aspartate (NMDA), GABA, SR95531 (Gabazine), DL-2-Amino-5-phosphonopentanoic acid DL-(AP5), L-(+)-2-Amino-4-phosphonobutyric acid (L-AP4) (Tocris Bioscience). Gramicidin (Sigma-Aldrich). DNQX was dissolved in dimethylsulphoxide (DMSO). The final concentration of DMSO in the bathing solution was 0.1%. At this concentration, DMSO did not modify the shape or the kinetics of synaptic currents. Drugs were applied in the bath *via* a three-way tap system, by changing the superfusion solution to one differing only in its content of drug(s). The ratio of flow rate to bath volume ensured full exchange within 2min. Values are mean ± SEM.

In vivo LTP

These experiments were carried out on adult (4 to 5 months old) males. Animals were allowed *ad libitum* access to chow and water. Eight animals were used for each experimental group. Double-pulse and long-term potentiation (LTP) studies were carried out on eight additional animals per group. Procedure for surgery, recordings and LTP induction were performed according to previous studies (Gruart et al., 2007).

Fear conditioning

Adult male mice (2-4 months of age) were trained and tested in an operant chamber (18.5 x18 x 21.5cm) (Coulbourn Instruments). The auditory cue (tone) emanated from a loudspeaker located in the sidewall, 15cm from the floor. The activity of animals was recorded directly on a computer through a single camera located at the top of the chamber. The presentation of tone and shock stimuli in all training and testing sessions was controlled by GraphicState software (Coulbourn Instruments). Mice were allowed to acclimate to the training chamber for 2min, and then a tone (CS) of 2800 Hz frequency and 85dB intensity was presented for 30sec and co-terminated in the last 2sec with a mild footshock (0.5 mA) [unconditioned stimulus (US)]. Two minutes later, another CS-US pairing was presented, and 30sec later the mice were returned to their home cages. For contextual fear memory, 24hs later mice were placed back into the same chamber under the same conditions, but this time no tone or foot shock was applied. The time the animals spent without moving for more than 2sec (freezing behaviour) was measured. Twenty-four hours after the contextual test, mice were tested for cued conditioning in a chamber with altered appearance and odour. The test lasted 6min, with 2min habituation phase and two 1min CS-US presentations, spaced by 2min inter trial interval (ITI). Number of animals are reported in the figure legend.

Object-context recognition

This task was performed according to previously published protocols (Wilson et al., 2013). Details of experimental design are reported in Figure S7.

Histology and Immunohistochemistry (IHC)

Brain tissues were prepared as previously described (Geibel et al., 2014). Free-floating 30µm brain sections were used for histology and IHC. Sections were chosen from two or three animals for each genotype (as reported in the relevant figure legend) and gave similar results. Representative images are shown. For IF and 3,3'-diaminobenzidine-based immunostainings sections were treated as previously described (Geibel et al., 2014) and probed with specific antibodies (Primary antibodies: Calbindin, Sigma CB995, 1:400; chicken anti-GFP, Abcam (ab 13970), 1:3000; Calretinin, Swant 7699/4, 1:1000; Parvalbumin, Swant PV-25, 1:1000. Phospho-Creb (Ser133), Cell Signalling 87G3 1:1000. Ki67, Abcam ab 15580, 1:5000. DCX, Abcam ab113435, 1:250. Secondary antibodies: alexaFluor 555 donkey anti-rabbit, Invitrogen, 1:1000; alexaFluor 555 anti-mouse, Invitrogen, 1:1000; alexaFluor 488 donkey anti-chicken, Jackson Immunoresearch, 1:1000; biotinylated anti-rabbit Vector Labs (BA-1000), 1:200; biotinylated anti-mouse Vector Labs (BA_2000), 1:200.

Immunoblotting

Mice were sacrificed by cervical dislocation and hippocampi were dissected and snap frozen on dry ice. Tissues were homogenized in 10X volume RIPA buffer (50mM Tris, 150mM NaCl, 1% Triton X-100, 1mM EDTA, 0.1% SDS, 0.5% sodium deoxycholate) containing protease and phosphatase inhibitors (Thermo Scientific). After mild sonication (5min, cycle: 30sec On/Off- Bioruptor) the lysates were centrifuged at 4°C, 20000g for 20min to remove insoluble materials. The concentration of the lysates was measured using the Pierce™BCA Protein Assay Kit. Proteins lysates were treated with loading buffer (50mM Tris pH 6.8, 2%SDS,

10%glycine, 4M UREA and 100mM DL-Dithiothreitol (DTT)) at 70ºC for 10min, resolved on a 7% SDS-PAGE gel, and transferred to nitrocellulose filter membranes using the BioRAD apparatus. Membranes were blocked in 2% fish gelatine and 0.2% Tween in PBS for 1hr at room temperature and incubated with primary antibodies (TrkB, Cell Signalling #4603, 1:500; KCC2, Upstate 07-432, 1:5000; NKCC1, Sigma-Aldrich, T4-S, 1:500; GAPDH, Sigma #G954S, 1:10000) overnight at 4°C. After washes in PBS+ 0.1% Tween (PBS-T) for 30min, secondary antibody (Goat anti-rabbit HRP, Stratech Jackson, 1:5000; Donkey anti-goat HRP, Stratech Jackson, 1:5000; Goat anti-mouse rabbit HRP, Santa-Cruz, 1:5000) was applied for 1hr at room temperature in blocking buffer. The membranes were washed in PBS-T for 30min and developed using enhanced chemiluminescence (ECL, Santa Cruz) on Hyperfilm™ ECL (Amersham). To detect KCC2 oligomers, samples were resuspended in 50mM Tris pH 6.8, 2%SDS, 10%glycine, 4M UREA and 0.5% lithium dodecyl sulphate (LDS) to solubilise KCC2, in non-reducing conditions and warmed at 37°C for 10min. Proteins were resolved by linear 3-8% Tris-acetate gel system (NuPAGE, Life technologies) and immunoblotted as described above. WBs were performed on 6 animal per group except otherwise indicated in the figure legend. Similar results were obtained on 2 technical replicates and representative images are shown. Densitometry of immunoreactive bands was performed using the Image Studio Lite software (LI-COR sciences).

smFISH

Harvested postnatal day seven (P7) brains were embedded in OCT, frozen and stored at -80°C until used. smFISH was performed as described by Stellaris (LGC Biosearch Technologies, Petaluma, CA, USA) RNA FISH frozen tissue protocol, and Lyubimova A, *et al.* 2013 (Lyubimova et al., 2013). https://biosearchassets.blob.core.windows.net/assets/bti_stellaris_protocol_frozen_tissue.pdf with minor modifications. Probe libraries were designed to target the coding sequence of *Nkcc1* gene using Stellaris Probe Designer online tool. The library consisted of 48 probes of 20 bps each (Table S4). The probes were coupled to Quasar570 fluorophore. Cryosections (8µm) were collected and directly mounted onto coverslips. Sections were then fixed in 4% paraformaldehyde for 10min at room temperature, followed by 1hr permeabilization using 70% ethanol at room temperature. Hybridization with 250nM fluorescent labelled probes was carried out overnight at 37°C. Sections were counterstained with DAPI and mounted on slides with Vectashield antifade mounting medium (Vector Laboratories Ltd, Burlingame, CA, USA). Image stacks (0.2µm distance) were acquired with a Leica microscope equipped with a x100 oil-immersion objective and a Leica DFC 365 FX camera using Leica AF6000 software (Leica, Wetzlar, Germany). Images were processed by a 3D reconstruction software (Leica AF6000) followed by image projection. Three random fields in the CA3 region were imaged for each section (2 sections per mouse at CA3 Bregma level 1.95-2.15mm) for a total of 376 cells for control and 398 cells for mutants analysed from 3 mice per genotype. Similar results were obtained on 2 technical replicates and representative images are shown. Quantification of the mRNA dots was done by using StarSearch tool developed by Raj lab (http://rajlab.seas.upenn.edu). DAPI was used to count the number of cells in each field.

Electron microscopy

Tissue for transmission electron microscopy was prepared as previously described (Wright et al., 2010). Briefly, mice were transcardially perfused with saline solution (0.9% NaCl, 500 units heparin per 500ml solution), followed by 0.1M PB buffer (pH7.4, EM-grade) containing 4% paraformaldehyde and 2.5% glutaraldehyde as previously described. Brains were removed before immersion in fixative solution at room temperature for a further 2hrs. Coronal vibratome sections (1µm) containing the hippocampus were post-fixed in 1% osmium tetroxide in 0.1M PB for 45min before dehydration through an ascending series of ethanol solutions and propylene oxide. Brain sections were then embedded in Durcupan resin on glass slides. Regions of the hippocampus were then cut out from a randomly selected section using a scalpel and glued onto a resin block for sectioning. Ultrathin sections (~60/70nm) were cut and collected on formvar-coated grids (Agar Scientific, UK), stained with uranyl acetate and lead citrate in an LKB Ultrostainer and then quantitatively assessed in a Philips CM12 transmission electron microscope (TEM). Standard ultrastructural characteristics were used (Gillingwater et al., 2006) to classify synapses as either symmetrical or asymmetrical. Number of animals are reported in the figure legend.

Timm's stain

Young adult mice (2 months old, n=3 per group) were treated according to standard procedure. Briefly, mice were perfused transcardially with sulfide solution (150mM Na_2S, 60mM NaH_2PO_4) for 2min, then with glutaraldehyde solution (3% glutaraldehyde, 294mM KH_2PO_4, 1.2M Na_2HPO_4, pH 7.35) for 3min followed by sulfide solution for another 2min. Brains were fixed in 20% saccharose in glutaraldehyde solution for 24hrs at 4°C. Brains were frozen for 5min on dry ice, and 40µm cryosections were collected on gelatinized slides and dried at 37°C overnight. Slides were incubated in a developer solution (30% gum arabic, a few crystals of thymol, 121mM citric acid, 81mM tri-sodium citrate dihydrate, 154mM hydrochinon, 5mM $AgNO_3$) at 25°C for 30-45min, rinsed with water, dehydrated in 70%, 95%, 2x 100% ethanol, 2x xylol for 2min each and mounted with DEPEC Eukitt.

Golgi-Cox staining

Two months-old mice (Control: n=9 neurons from 4 mice; mutant: n=12 neurons from 3mice), were used for these experiments and treated according to a previously published protocol (Gibb and Kolb, 1998).

Dendritic branching and spine density analysis

Golgi stained neurons from the CA3 and DG hippocampal area were selected for analyses according to the following criteria: (i) full impregnation of the neurons with no apparent dendritic truncation; (ii) presence of at least two primary basilar and one apical dendrite, (iii) each dendrite branched at least once (dendrites arising from the cell body were considered as first-order until they bifurcated into second- order segments and so on). Selected neurons in the respective regions were traced with NeuroLucida, and reconstructed with the software program NeuroLucida Explorer (MicroBrightField Inc.) for automatic measurements of dendritic tree complexity using Sholl analysis. The dendrites complexity was examined by measuring the number of intersections made by dendrites with concentric circles (Sholl rings) drawn at progressive distances (25 µm) from the soma. On selected neurons in CA3 and DG,

the number of dendritic spines was manually counted with the support of NeuroLucida software. Only protrusions with a clear connection of the head of the spine to the shaft of the dendrite were counted as spines. Dendritic spine density was expressed as the ratio number of spines/lengths of the dendritic segment, except first order ones. An experimenter blind to experimental groups performed all analyses.

Statistical analysis

For the *in vitro* electrophysiology recordings in neonatal animals, data were sampled at 20 kHz and filtered with a cut-off frequency of 2 kHz. Data acquisition was made using pClamp 9 (Axon Instruments, Foster City, CA). Spontaneous and miniature GABAergic and glutamatergic synaptic currents were analysed offline with Clampfit 9 program. They were first collected using the template function of Clampfit and then reviewed by visual inspection. The significance of differences was assessed (using the GraphPad Prism 7) by unpaired Student's *t*-test. A non-parametric test (Wilcoxon signed-rank test) was used when data did not pass the normality test. In adult animals, statistical comparisons were performed by either unpaired Student's *t*-test or one-way ANOVA. For the *in vivo* LTP experiments, statistical analysis was performed using the SPSS 13.0 for Windows package (SPSS Inc, Chicago, IL). Data were analysed by two-way ANOVA repeated measure (One Factor Repetition). For the OF, OCR tasks, statistical analysis was performed using GraphPad Prism 6. Mean distance in the OF was analysed using two-tailed Student's *t*-test, while habituation to the arena and anxiety in the OF was assessed by two-way ANOVA repeated-measures. Discrimination ratio in the OCR was analysed by two-tailed unpaired Student's *t*-test. Total object exploration was assessed by two-way ANOVA during the acquisition phase, and by two-tailed unpaired Student's *t*-test during the test phase. For the FC, the time freezing during the conditioning phase and the cued FC test were analysed by two-way ANOVA repeated-measures, followed by Sidak's post-hoc analyses. Two-tailed unpaired Student's t-test was used to examine the percentage of time spent freezing evoked by the context. For the immunoblots and the EM, statistical significance was assessed using the unpaired Student's *t*-test. Dendritic length, number of intersections and spine density from Golgi stained neurons were analysed using unpaired Student *t*-test. The significance level (alpha) for all tests was set at 0.05, and P values were considered significant when $P<0.05$. More details for statistical tests are indicated in the figure legends. Datasets for statistical analysis for the following figures (Figure S6A-B, Figure 6B-C, Figure S6D-E) are attached as Tables (S1-3).

SUPPLEMENTAL REFERENCES

Berg, D.A., Su, Y., Jimenez-Cyrus, D., Patel, A., Huang, N., Morizet, D., Lee, S., Shah, R., Ringeling, F.R., Jain, R.*, et al.* (2019). A Common Embryonic Origin of Stem Cells Drives Developmental and Adult Neurogenesis. Cell *177*, 654-668 e615.

Geibel, M., Badurek, S., Horn, J.M., Vatanashevanopakorn, C., Koudelka, J., Wunderlich, C.M., Brönneke, H.S., Wunderlich, F.T., and Minichiello, L. (2014). Ablation of TrkB signalling in CCK neurons results in hypercortisolism and obesity. Nat Commun *5*, 3427.

Gibb, R., and Kolb, B. (1998). A method for vibratome sectioning of Golgi-Cox stained whole rat brain. J Neurosci Methods *79*, 1-4.

Gillingwater, T.H., Ingham, C.A., Parry, K.E., Wright, A.K., Haley, J.E., Wishart, T.M., Arbuthnott, G.W., and Ribchester, R.R. (2006). Delayed synaptic degeneration in the CNS of Wlds mice after cortical lesion. Brain *129*, 1546-1556.

Gruart, A., Sciarretta, C., Valenzuela-Harrington, M., Delgado-Garcia, J.M., and Minichiello, L. (2007). Mutation at the TrkB PLC{gamma}-docking site affects hippocampal LTP and associative learning in conscious mice. Learn Mem *14*, 54-62.

Lyubimova, A., Itzkovitz, S., Junker, J.P., Fan, Z.P., Wu, X., and van Oudenaarden, A. (2013). Single-molecule mRNA detection and counting in mammalian tissue. Nat Protoc *8*, 1743-1758.

Madisen, L., Zwingman, T.A., Sunkin, S.M., Oh, S.W., Zariwala, H.A., Gu, H., Ng, L.L., Palmiter, R.D., Hawrylycz, M.J., Jones, A.R.*, et al.* (2010). A robust and high-throughput Cre reporting and characterization system for the whole mouse brain. Nat Neurosci *13*, 133-140.

Minichiello, L., Korte, M., Wolfer, D., Kühn, R., Unsicker, K., Cestari, V., Rossi-Arnaud, C., Lipp, H.P., Bonhoeffer, T., and Klein, R. (1999). Essential role for TrkB receptors in hippocampus-mediated learning. Neuron *24*, 401-414.

Novak, A., Guo, C., Yang, W., Nagy, A., and Lobe, C.G. (2000). Z/EG, a double reporter mouse line that expresses enhanced green fluorescent protein upon Cre-mediated excision. Genesis *28*, 147-155.

Ohtsuka, N., Badurek, S., Busslinger, M., Benes, F., Minichiello, L., and Rudolph, U. (2013). GABAergic neurons regulate lateral ventricular development via transcription factor Pax5. Genesis *51(4):234-45.*

Srinivas, S., Watanabe, T., Lin, C.S., William, C.M., Tanabe, Y., Jessell, T.M., and Costantini, F. (2001). Cre reporter strains produced by targeted insertion of EYFP and ECFP into the ROSA26 locus. BMC Dev Biol *1*, 4.

Wilson, D.I., Langston, R.F., Schlesiger, M.I., Wagner, M., Watanabe, S., and Ainge, J.A. (2013). Lateral entorhinal cortex is critical for novel object-context recognition. Hippocampus *23*, 352-366.

Wright, A.K., Wishart, T.M., Ingham, C.A., and Gillingwater, T.H. (2010). Synaptic protection in the brain of WldS mice occurs independently of age but is sensitive to gene-dose. PLoS One *5*, e15108.
